# Source Sector Mitigation of Solar Energy Generation Losses Attributable to Particulate Matter Pollution

**DOI:** 10.1021/acs.est.2c01175

**Published:** 2022-06-01

**Authors:** Fei Yao, Paul I. Palmer

**Affiliations:** † School of GeoSciences, University of Edinburgh, Edinburgh EH9 3FF, U.K.; ‡ National Centre for Earth Observation, University of Edinburgh, Edinburgh EH9 3FF, U.K.

**Keywords:** Particulate matter, Photovoltaics, GEOS-Chem, PVLIB-Python, Emissions

## Abstract

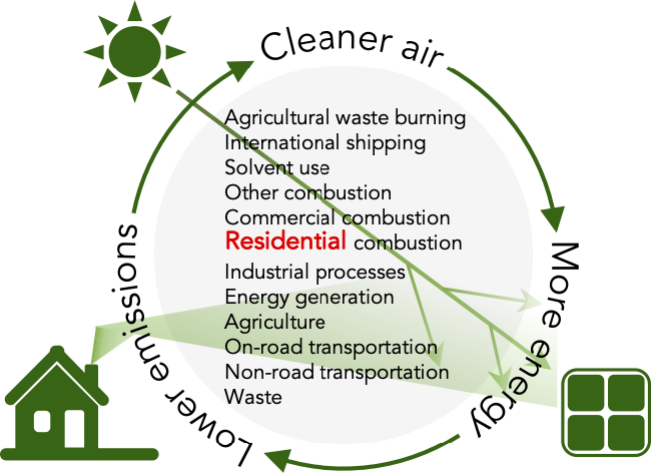

Particulate matter (PM) in the atmosphere and deposited on solar photovoltaic (PV) panels reduce PV energy generation. Reducing anthropogenic PM sources will therefore increase carbon-free energy generation and as a cobenefit will improve surface air quality. However, we lack a global understanding of the sectors that would be the most effective at achieving the necessary reductions in PM sources. Here we combine well-evaluated models of solar PV performance and atmospheric composition to show that deep cuts in air pollutant emissions from the residential, on-road, and energy sectors are the most effective approaches to mitigate PM-induced PV energy losses over East and South Asia, and the Tibetan Plateau, Central Asia, and the Arabian Peninsula, and Western Siberia, respectively. Using 2019 PV capacities as a baseline, we find that a 50% reduction in residential emissions would lead to an additional 10.3 TWh yr^–1^ (US$878 million yr^–1^) and 2.5 TWh yr^–1^ (US$196 million yr^–1^) produced in China and India, respectively.

## Abstract 2

We quantify benefits to photovoltaic energy generation from reducing emissions in individual anthropogenic sectors that helps to prioritize emission mitigation strategies.

## Introduction

Our harnessing energy provided for free by the sun has a low environmental footprint and will therefore play a role in reducing emissions of greenhouse gases and mitigating the harmful impacts of climate change.^1,2^ A variety of technologies convert sunlight to usable electricity, but currently the most common approach is to use solar photovoltaic (PV) panels. The past decade (2011 to 2020) has seen an enormous increase in the worldwide solar PV installed capacity, from 72 to 707 GW.^3^ Solar PV is expected to dominate growth in the renewable energy sector for the foreseeable future.^4^ However, particulate matter (PM), a mixture of solid particles and liquid droplets suspended in the air, represents a major barrier to maximizing the performance of solar PV technologies and therefore compromises our ability to generate clean energy. Atmospheric PM scatters and absorbs the solar radiation that would otherwise reach the solar panels.^5−7^ PM deposited on the solar panels further impedes the solar radiation being received by the PV semiconductor material.^8−10^

PM is released directly into the atmosphere via processes such as combustion (primary source) and is formed in the atmosphere from the condensation of low-volatility gases (secondary source).^11,12^ These primary and secondary sources of PM are emitted from a wide range of anthropogenic activities. Reductions in these emissions are required to improve the energy generation performance of solar PV cells,^7,13^ but it remains unclear which are the most effective source sectors to target. Natural sources of PM can also be significant on a regional basis^14,15^ but are not easily controlled and therefore are not the subject of this study.

To calculate the benefits of stringent 50% global emission reductions from individual source sectors to solar PV electricity generation, we integrate the GEOS-Chem global 3-D model of atmospheric composition, equipped with online radiative transfer calculations, with PVLIB-Python, which is a solar PV performance model. We focus on source sectors to identify a systematic approach to prioritizing mitigation measures.^16^ The 50% source sector reductions we explore are large but feasible, with success being previously reported in the US^17^ and China.^18^ Following previous studies,^7,10,19,20^ we use capacity factors (CFs) to describe solar PV electricity generation efficiency. We calculate CF values as the ratio of the actual power output of a PV panel to the theoretical maximum power output.

Our experimental design enables us to distinguish PV efficiency losses due to atmospheric and deposited PM, which we term as PM dimming and soiling impacts, respectively. Conversely, abatements in PM dimming and soiling impacts resulting from emission reductions are called brightening and cleaning benefits, respectively. We report our results for three widely used panel settings: horizontal fixed (flat), fixed with optimal tilt (tilt), and one-axis tracking (OAT). We describe their characteristics in Table S1.

## Materials and Methods

Figure S1 describes how we integrate v12.9.3 of the GEOS-Chem global 3-D model of atmospheric composition^21^ equipped with the rapid radiative transfer model for general circulation models (GCMs),^22^ a configuration known as GCRT,^23^ with v0.8.0 of the PVLIB-Python model, a solar PV performance model,^24^ to estimate PV efficiency.

Briefly, the GCRT simulates PM dry mass concentrations, computes global horizontal irradiance (GHI) under control and no-PM conditions, and provides PM gravitational and turbulent deposition velocities.^25^ To calculate the irradiance reaching the solar panels and subsequently the solar cells, we combine the GCRT model outputs with surface albedo and precipitation rates (*p*) from MERRA-2 (modern-era retrospective analysis for research and applications, version 2) meteorological analyses^26^ and with solar positions and solar panel configurations determined by the PVLIB-Python model. Using the PVLIB-Python model, we calculate PV efficiency from the irradiance received by the solar cells together with ambient temperature and wind speed from MERRA-2 meteorological analyses. With our integrated model approach, we are able to investigate the impact of reducing emissions and sweeping panels on PV efficiency.

### GEOS-Chem Model Coupled with the Rapid Radiative Transfer Model for GCMs

We configure the GCRT, driven by MERRA-2 meteorological analyses, to provide 3 hly output at a horizontal resolution of 2° (latitude) × 2.5° (longitude) from July 2005 through December 2017, the first 2.5 years of which serve as the model spin-up period. We set the atmospheric transport and chemistry time steps to 10 and 20 min, respectively. We use 47 hybrid-σ levels from the surface to 0.01 hPa, of which 30 lie below the dynamic tropopause.

The Community Emissions Data System, updated for the Global Burden of Disease - Major Air Pollutant Sources project (CEDS_GBD-MAPS_),^27^ provides the most contemporary global emission estimates to date for 7 major atmospheric pollutants as a function of 11 detailed emission source sectors: agriculture (noncombustion sources only), energy generation, industrial processes, nonroad and on-road transportation, separate residential, commercial, and other sectors, waste, solvent use, and international shipping. CEDS_GBD-MAPS_ does not include emissions from agricultural waste burning, which is also an important anthropogenic PM source and is available through the fourth version of the Global Fire Emissions Database (GFED).^28^ In this study, we use the original CEDS_GBD-MAPS_ and GFED emissions to perform control simulations (CTRL), while we halve emissions of all atmospheric pollutants sector by sector to perform mitigation simulations (0.5SECTOR). Both control and mitigation simulations are representative of the 2008–2017 period. In addition to the CEDS_GBD-MAPS_ and GFED anthropogenic emissions, we provide both control and mitigation simulations with necessary, identical emissions from aircraft,^29^ open fires from tropical deforestation, boreal forest, peat, savannah, and temperate forest,^28^ soil^30^ and lightning^31^ nitrogen oxides, biogenic volatile organic compounds^32^ and ammonia,^33^ volcanic sulfur dioxide,^34^ mineral^35^ and anthropogenic^36^ dust, oceanic sea salt,^37,38^ and the remaining emission sources.^39−47^

GCRT includes a detailed NO_*x*_–O_*x*_–hydrocarbon–aerosol–bromine–chlorine–iodine chemical mechanism applied from the surface to the tropopause.^21^ The sulfate (SO_4_)–nitrate (NIT)–ammonium (NH_4_) secondary inorganic aerosol (SNA) was developed in ref (48) with thermodynamics being computed by the ISORROPIA thermodynamic module.^49^ The organic aerosol (OA) simulation follows the simple, irreversible, direct yield scheme of ref (50). We assume an OA to organic carbon (OC) mass ratio of 2.1.^51^ Simulations of black carbon (BC), dust, and sea salt are described in refs (38), (52, and 53), respectively.

We use GCRT to simulate dry mass concentrations of a total of 17 PM species: SO_4_, NIT, NH_4_, hydrophilic (OCPI) and hydrophobic (OCPO) OC, secondary OA (SOA), hydrophilic (BCPI) and hydrophobic (BCPO) BC, accumulation (SALA) and coarse (SALC) mode sea salt, and dust distributed in seven size bins.^54^ GHIs computed under control and no-PM conditions are used to determine PM dimming impacts. PM dry deposition velocities^25^ are output and combined with PM dry mass concentrations to determine PM dry mass fluxes on solar panels. Combining PM dry mass fluxes with precipitation and elapsed time (relative to 00:00:00 UTC January 1, 2008) helps to determine the accumulated PM dry mass per unit area deposited on solar panels, as detailed below.

### Linking GCRT to the PVLIB-Python Model

We estimate the direct normal irradiance (DNI) and the diffuse horizontal irradiance (DHI) from GHI using the Erbs model.^55^ We calculate the irradiance transposed to the solar panels, defined as “in” plane-of-array irradiance, POAI_in_, by summing the beam, ground-reflected, and sky-diffuse components of POAI_in_. They are derived from DNI, GHI, and DHI, respectively, on the basis of solar positions (i.e., solar zenith and azimuth) and solar panel configurations (i.e., tilt angle and azimuth of solar panels)^56^ determined by the PVLIB-Python model.

PM deposited on solar panels further reduces POAI_in_ to “out” plane-of-array irradiance, POAI_out_, which is the incident irradiance reaching the solar cells where electricity conversion occurs. An intuitive way linking POAI_in_ to POA_out_ is to use the broad-band-wise optical depth (τ) of deposited PM: i.e., POAI_out_ = POAI_in_ × e^–τ^. Following refs (9 and 10), we determine τ from the accumulated dry mass per unit area and the measured optical properties of each deposited PM species: i.e., τ = ∑_*i* = 1_^17^((*E*_abs,i_ + β_i_*E*_scat,i_) × PM_i_), where i denotes a deposited PM species for which the absorption and scattering mass extinction coefficients, the backscattering ratio, and the accumulated dry mass per unit area are termed *E*_abs,i_ and *E*_scat,i_, β_i_, and PM_i_, respectively. We give values of *E*_abs,i_, *E*_scat,i_, and β_i_ in Table S2. In our work (not shown), we also explore using the wavelength-dependent PM mass extinction coefficients, taken from the lowest GCRT model layer where solar panels are located, to calculate τ. However, this generally leads to a larger estimate for τ and consequently a higher estimate for PM soiling in comparison to prior studies.^9,10^ The underlying reasons include the difficulty and the uncertainty of compiling multiple wavelength-dependent PM mass extinction coefficients into a broad-band-wise estimate and that deposited PM will likely have different absorption and scattering properties in comparison to atmospheric PM due to the different environment. Hence, we recommend further empirical studies to use and expand the available measured optical properties of deposited PM. There is also a need to consider the possible influence of the mixing of deposited PM, as can commonly occur, on its optical properties.

PM_i_ is the net combination of PM accumulation and removal processes occurred on solar panels: i.e., PM_i_ = PM_i_^Accum^ – PM_i_^Removal^. PM_i_^Accum^ comes from PM dry deposition processes. PM_i_^Accum^=∫_*t*_(*V*_i_^g^ cos θ_T_ + *V*_i_^t^)*C*_i_ d*t*, where *V*_i_^g^ and *V*_i_^t^ are gravitational and turbulent dry deposition velocities for PM species i, respectively. *C*_i_, extracted from the lowest GCRT model layer where solar panels are located, is the surface dry mass concentration for PM species i. The gravitational velocity is vertical; thus, we reduce it on tilted solar panels by multiplying cos θ_T_, where θ_T_ is the tilt angle of solar panels.

PM_i_^Removal^, based on *p*, which shows low bias, high correlation, and a realistic diurnal cycle in comparison with observations from the Global Precipitation Climatology Project version 2.2,^57^ follows ref (10):

- When *p* ⩽ 1 mm h^–1^, no PM removal occurs.
- When 1 < *p* ⩽ 3 mm h^–1^, SNAs are entirely removed and half of the OAs are removed.
- When 3 < *p* ⩽ 5 mm h^–1^, SNAs are entirely removed and half of all other PMs are removed.
- When *p* > 5 mm h^–1^, all PMs are removed.

### PVLIB-Python Model

The PVLIB-Python model is a community-supported tool that provides a set of functions and classes for simulating the performance of solar PV energy systems. PVLIB-Python v0.8.0 currently supports performance modeling of flat, tilt, and OAT panels (Table S1).

For each panel, the PVLIB-Python model applies different solar panel configurations to transpose solar radiative fluxes to irradiance received by the solar panels, POAI_in_. The PVLIB-Python model takes the POAI_out_ reduced from POAI_in_ and ambient temperature and wind speed from MERRA-2 meteorological analyses as inputs to calculate the cell temperature and effective irradiance,^58^ further uses the PV module (Canadian_Solar_CS5P_220M___2009_) to calculate the direct (DC) power,^58^ and finally applies the inverter (ABB__MICRO_0_25_I_OUTD_US_208__208V_) to calculate the alternating (AC) power.^59,60^

In this process, both the PV cell efficiency (solar energy to DC power, 12.94%) and the inverter efficiency (DC to AC power, 96%) are considered. We model a single module that contains 96 cells in series and ignore potential electricity losses due to, for example, degradation of modules and inverters. We use this approach in the absence of an established model describing these losses. We divide the AC power by the AC power rating of the inverter (250 W) to obtain CF that describes PV efficiency.

### Model Evaluation

We evaluate the integrated model against a range of *in situ* observations. We describe the methods, results, implications, and limitations of our model evaluation in Text S1 and Figures S2–S5. We find that the integrated model can generally reproduce the observed variations in GHI and levels of atmospheric and deposited PM during both periods of high and low solar insolation. This provides us with confidence in our use of the integrated model to identify the main sources of PM pollution affecting PV power output.

### Experimental Design

A perturbation to upstream emissions will simultaneously affect PM dimming and soiling processes. We therefore calculate three CFs: (1) CF1 includes both PM dimming and soiling impacts, namely real PV efficiency, (2) CF2 includes only PM dimming impacts, and (3) CF3, which does not include PM dimming or soiling impacts. This allows us to isolate PM dimming (CF3 – CF2), soiling (CF2 – CF1), and total (CF3 – CF1) impacts. We determine brightening, cleaning, and total benefits from halving source sector emissions by taking the difference in each of these quantities between the control and mitigation simulations: i.e., (CF3 – CF2)_CTRL_ – (CF3 – CF2)_0.5SECTOR_, (CF2 – CF1)_CTRL_ – (CF2–CF1)_0.5SECTOR_, and (CF3 – CF1)_CTRL_ – (CF3–CF1)_0.5SECTOR_. Comparing these difference quantities across source sectors identifies which are the most effective to target the alleviation of PM-induced PV efficiency losses.

By simultaneously halving emissions across all geographical regions, we cannot explicitly investigate the role of long-range or regional transport on local PM pollution levels and PV power output.^61,62^ As the implementation of emission mitigation strategies is typically constrained to political borders, we need to consider the influence of upwind sources on the effectiveness of specific local policies that will also affect downwind PM pollution levels and PV power output.

Unlike reducing emissions, sweeping panels either manually or by robots affects only the PM soiling process. We evaluate the benefits of sweeping panels by comparing CF1s in the control simulation: i.e., CF1_CTRL+SWEEPING_ – CF1_CTRL_. We stipulate that all PM_i_s become zeros at the beginning of each year, quarter, month, week, and day to correspond to CF1_CTRL+SWEEPING_ of sweeping panels at yearly, quarterly, monthly, weekly, and daily frequencies, respectively.

## Results and Discussion

Throughout this study, we analyze the decadal (and corresponding seasonal) mean (2008–2017) PV efficiency, PM impacts, and benefits from reducing emissions and sweeping panels at each 2° (latitude) × 2.5° (longitude) grid for their spatial distributions and further calculate the regional area-weighted mean values for regional characteristics (and interannual variabilities). We use the latest IPCC climate reference regions^63^ (Figure S6) for our regional synthesis.

### PV Efficiency and PM Impacts

**Figure 1:**
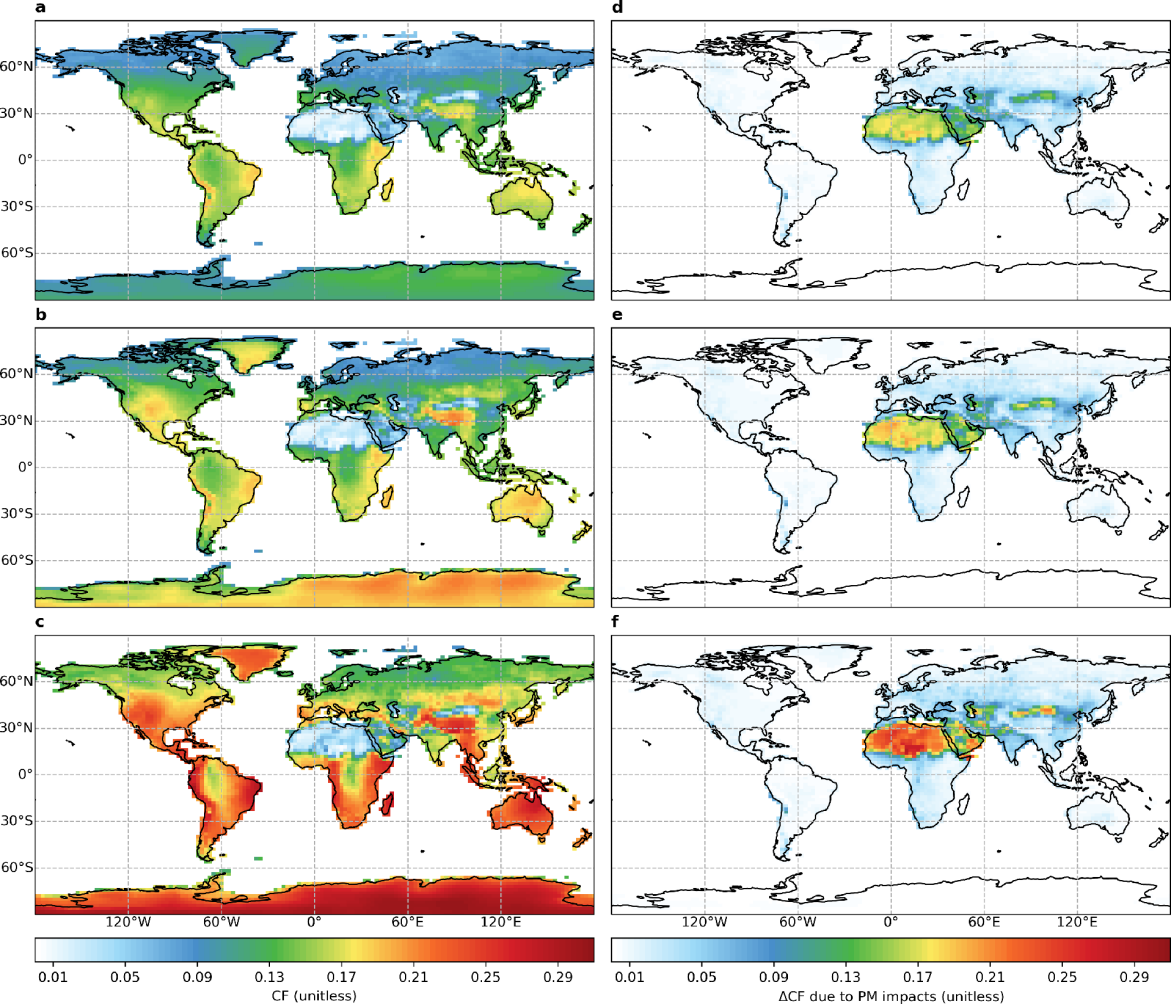
Geographical distributions of decadal mean (2008–2017) (a–c) PV efficiency and (d–f) its losses due to atmospheric and deposited PM for (a, d) flat, (b, e) tilt, and (c, f) one-axis tracking panels.

Figure 1 shows the geographical distributions of decadal mean (2008–2017) PV efficiency and its losses due to atmospheric and deposited PM for flat, tilt, and OAT panels. Globally, flat panels have an area-weighted mean CF of 0.12, with high values distributed over North and South America, Eastern and Southern Africa, the Tibetan Plateau, Southeast Asia, Australia, and Madagascar. Tilt panels improve the global area-weighted mean CF to 0.13 by enhancing values over high latitudes, particularly Greenland and Antarctica, where CFs are improved by more than 40%. OAT panels further improve the global area-weighted mean CF to 0.18, with similar value enhancements everywhere.

Regions with low CFs are typically associated with high PM impacts (Figure 1a–c versus Figure 1d–f). This is supported by the statistically significant (*p* < 0.05) negative Pearson correlation coefficients between regional area-weighted mean PV efficiency and PM impacts for flat (−0.61), tilt (−0.72), and OAT (−0.73) panels (Figure S7). We find similar spatial distributions of PM impacts (Figure 1d–f), with OAT panels having the largest values, followed by tilt and flat panels. Desert regions including the Sahara, Arabian Peninsula, and Central Asia report PM impacts that are comparable to the maximum PV efficiency achieved elsewhere (e.g., ∼0.26 in OAT panels).

**Figure 2:**
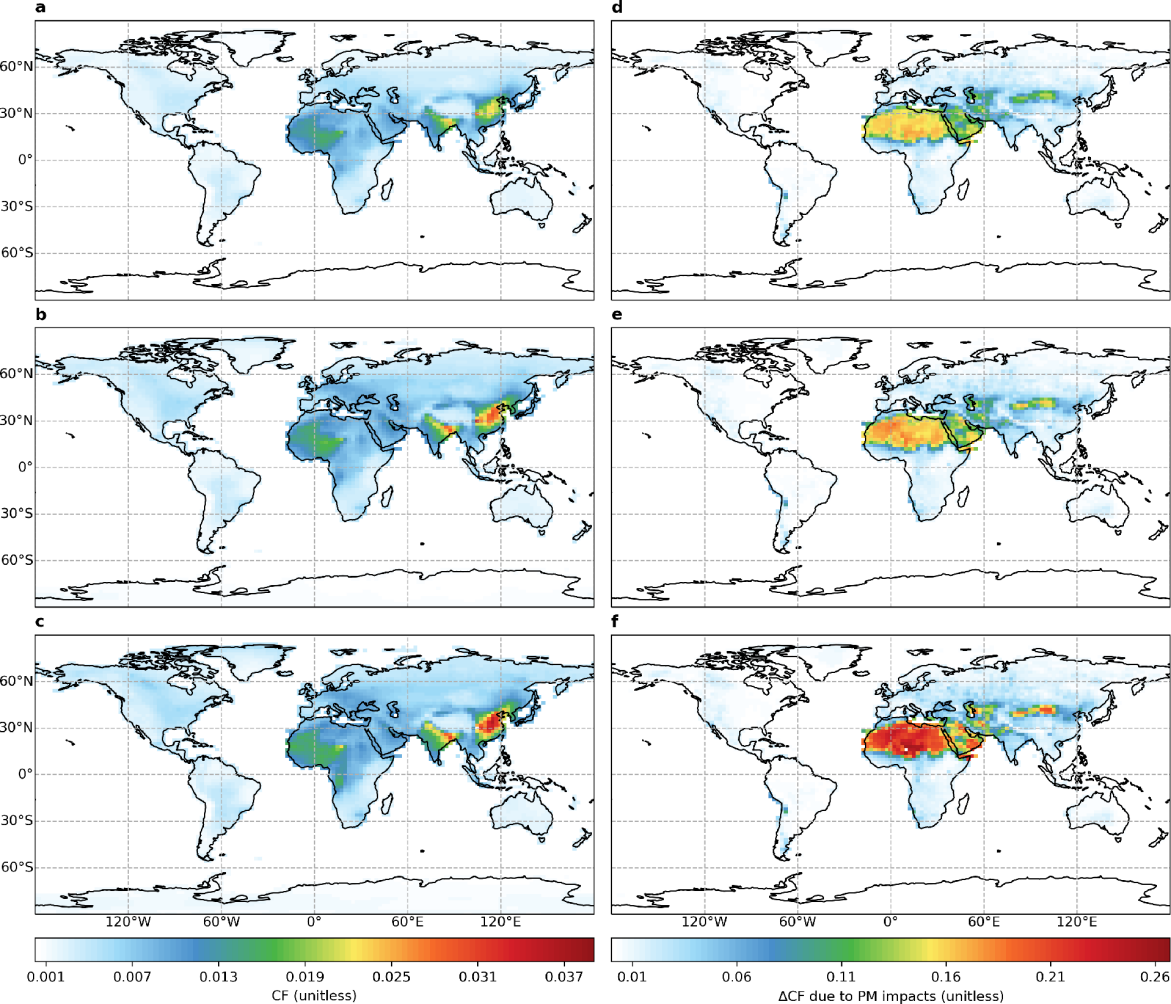
Geographical distributions of decadal mean (2008–2017) PV efficiency losses due to (a–c) atmospheric and (d–f) deposited PM for (a, d) flat, (b, e) tilt, and (c, f) one-axis tracking panels.

Figure 2 shows that by separating the total PM impact into dimming and soiling, we find that the magnitude and distribution of the total impact is almost exclusively determined by soiling, as the spatial pattern of the former largely follows that of the latter. The maximum magnitude of PM soiling impacts is almost 7 times that of PM dimming impacts. The strongest PM soiling impacts are over desert regions, a result of the rapid accumulation of dust (Figure S8) deposited on the solar panels and of limited removal by precipitation (Figure S9). Nonetheless, we find statistically significant (*p* < 0.05) positive Pearson correlation coefficients between regional area-weighted mean PM dimming and soiling impacts for flat (0.69), tilt (0.65), and OAT (0.61) panels (Figure S10). This suggests that PM dimming and soiling impacts are generally coincident so that decreasing emissions will help to reduce them simultaneously.

We find similar spatial patterns for PM dimming impacts across the three panel settings (Figure 2a–c), with larger values being found for OAT than tilt or flat panels. The main regions where PM dimming impacts are as high as 0.04 are East and South Asia, particularly over highly polluted regions such as North China and the Indo-Gangetic Plain, consistent with previous studies.^5,10^ Other regions where PM dimming impacts are moderate at ΔCF levels of 0.01 include West and Central Africa.

### Benefits of Reducing Emissions

Here we explore the extent to which we can reduce PM impacts, as described above, by decreasing PM emissions. We quantify and determine the maximum benefits of halving emissions from all anthropogenic source sectors.

**Figure 3:**
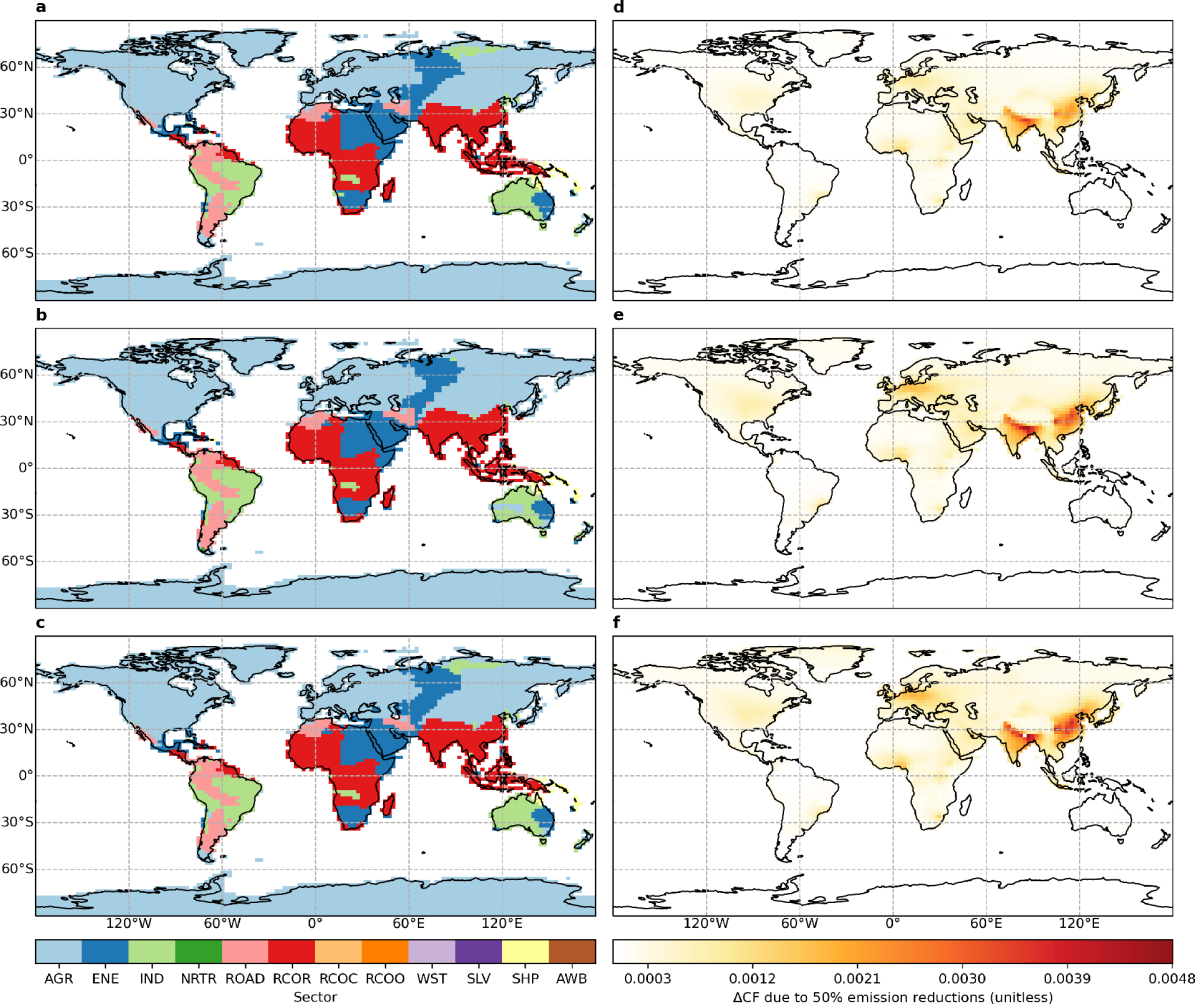
Geographical distributions of (a–c) source sectors from which halving emissions provides (d–f) maximum decadal mean (2008–2017) brightening benefits for (a, d) flat, (b, e) tilt, and (c, f) one-axis tracking panels. Full definitions for source sectors are noncombustion agriculture (AGR), energy generation (ENE), industrial processes (IND), nonroad (NRTR) and on-road (ROAD) transportation, separate residential (RCOR), commercial (RCOC), and other (RCOO) sectors, waste (WST), solvent use (SLV), international shipping (SHP), and agricultural waste burning (AWB).

Figure 3 and Table S3 show that halving residential and agricultural emissions result in widespread decreases in PM dimming. The proportions of areas occupied by the residential sector from which halving emissions provides the largest brightening benefits for flat, tilt, and OAT panels are 46%, 58%, and 50% and uniformly 93% over East and South Asia, respectively, and they are 43%, 37%, and 40% and uniformly 100% by the agricultural sector over East Asia and Western and Central Europe, respectively. The brightening benefits for the three panels of halving residential emissions are 8%, 9%, and 9% and equally 12% over East and South Asia, respectively, and they are equally 8% and equally 13% of halving agricultural emissions over East Asia and Western and Central Europe, respectively.

Seasonal statistics (Table S3) show that, over East Asia, halving residential, agricultural, industrial, and agricultural emissions results in the most widespread brightening benefits during DJF, MAM, JJA, and SON, respectively. The proportions of areas occupied by these four sectors from which halving emissions provide the largest brightening benefits for the three panels during the four seasons are in the ranges 99%, 79–80%, 68–72%, and 55–56%, respectively. The brightening benefits for the three panels from emission cuts to these four sectors during the four seasons are in the ranges 14–15%, 10%, 10–11%, and 9–10%, respectively. Despite the seasonal nature (e.g., heating from November to March in the north of China) of emissions from the residential sector, the large and widespread brightening benefits by halving emissions from this sector during DJF dominate the annual results.

The seasonal results (Table S3) of halving emissions over South Asia and Western and Central Europe are less complicated than those over East Asia. Except during JJA, when there are significant brightening benefits from halving energy emissions, halving residential and agricultural emissions consistently dominates the brightening benefits over South Asia and Western and Central Europe, respectively, throughout the year. The largest brightening benefits for the three panels vary 15–16% for South Asia during DJF and 15% for Western and Central Europe during SON.

**Figure 4:**
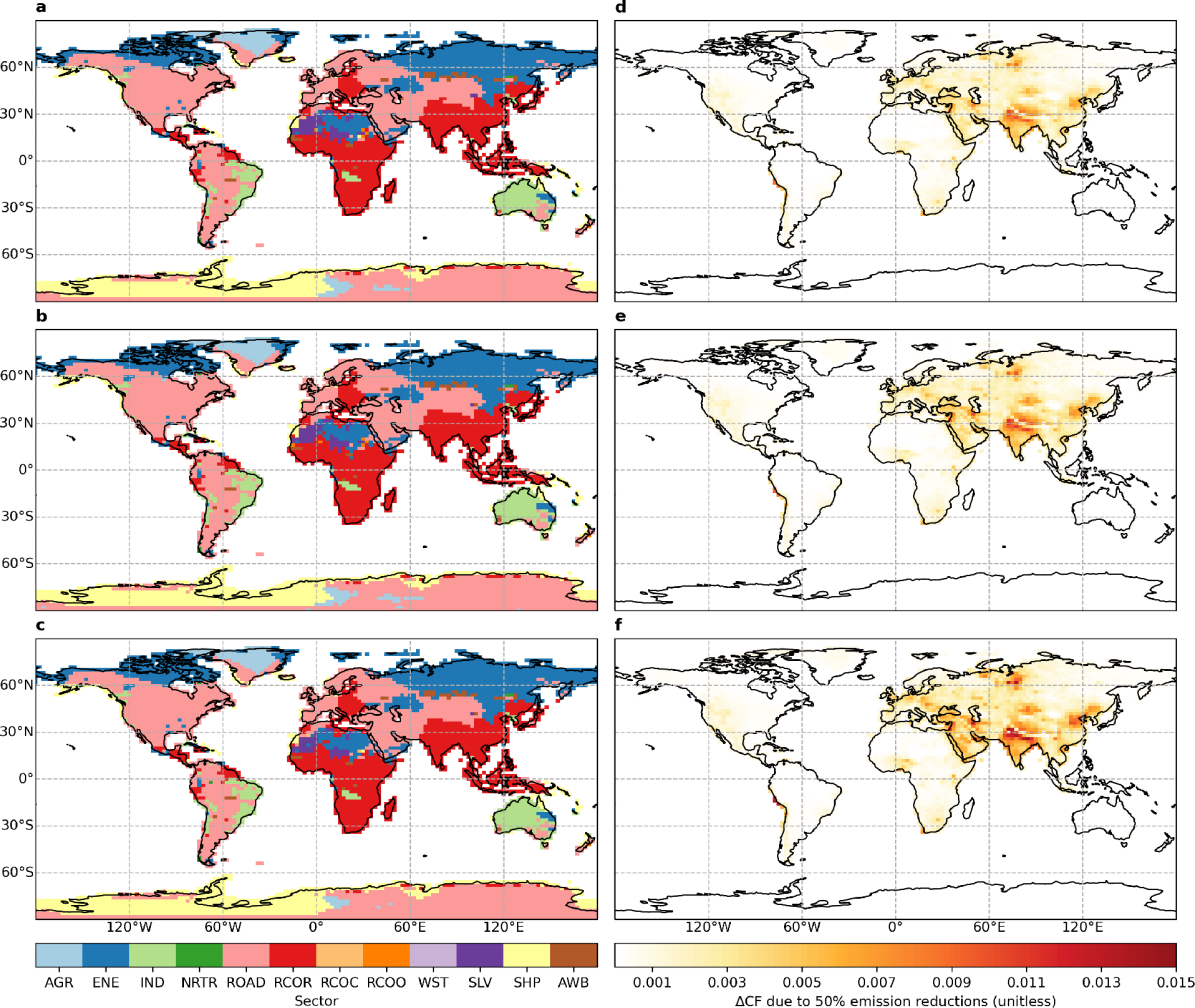
Geographical distributions of (a–c) source sectors from which halving emissions provides (d–f) maximum decadal mean (2008–2017) cleaning benefits for (a, d) flat, (b, e) tilt, and (c, f) one-axis tracking panels. Full definitions for source sectors are noncombustion agriculture (AGR), energy generation (ENE), industrial processes (IND), nonroad (NRTR) and on-road (ROAD) transportation, separate residential (RCOR), commercial (RCOC), and other (RCOO) sectors, waste (WST), solvent use (SLV), international shipping (SHP), and agricultural waste burning (AWB).

Figure 4 and Table S4 show that halving residential, on-road, and energy emissions result in widespread decreases in PM soiling. The proportions of areas occupied by the residential sector from which halving emissions provides the largest cleaning benefits for flat, tilt, and OAT panels are 90%, 90%, and 89%, uniformly 93%, and uniformly 91% over East Asia, South Asia, and the Tibetan Plateau, respectively, and they are 67%, 72%, and 74%, uniformly 87%, and uniformly 78% by the on-road sector over eastern Central Asia, western Central Asia, and the Arabian Peninsula, respectively. The corresponding values are uniformly 52% by the energy sector over Western Siberia. The cleaning benefits for the three panels of halving residential emissions are equally 12–13% over East and South Asia. The corresponding values are slightly higher at 15–17% over the Tibetan Plateau. The cleaning benefits for the three panels of halving on-road emissions are equally 2–4% over Central Asia and the Arabian Peninsula, and they are equally 10% of halving energy emissions over Western Siberia.

Seasonal statistics (Table S4) show that, over East Asia, the proportion of areas occupied by the residential sector from which halving emissions provides the largest cleaning benefits for the three panels follows a descending order of DJF, MAM, JJA, and SON, which are 96%, 93–94%, 65%, and 51–53%, respectively. The cleaning benefits for the three panels from emission cuts to the residential sector during DJF and MAM (12–13%) are slightly higher than those during JJA and SON (9–11%). The industrial sector is another place from which halving emissions provides significant cleaning benefits of 6% and 8–9% for the three panels during JJA and SON, respectively.

Table S4 also shows that halving residential emissions consistently dominates the cleaning benefits for the three panels over South Asia and the Tibetan Plateau throughout the year, with the largest values found during DJF, when they are 13–14% for South Asia and 16–18% for the Tibetan Plateau. Halving on-road emissions consistently dominates the approximately aseasonal cleaning benefits of 2–4% for the three panels over Central Asia and the Arabian Peninsula throughout the year. Halving energy emissions consistently dominates the cleaning benefits for the three panels over Western Siberia throughout the year, with the values during MAM and JJA (10–11%) being slightly larger than those during SON and DJF (7–9%).

The combined benefits from brightening and cleaning (Figure S11 and Table S5) mainly follow the pattern of cleaning benefits, as expected. On the decadal time scale, we report that halving residential emissions results in total benefits of 10–12% for the three panels over East and South Asia. The corresponding values are slightly higher at 15–16% over the Tibetan Plateau. Halving on-road emissions results in total benefits of 2–4% for the three panels over Central Asia and the Arabian Peninsula. Halving energy emissions results in total benefits of 9–10% for the three panels over Western Siberia.

### Impact on Energy Sector

The solar energy industry in East and South Asia stands to reap considerable rewards from halving residential emissions. To illustrate our point, we collect the installed PV capacities as of 2019 from Chinese^64^ and Indian^65^ national energy-related administration and combine them with our decadal mean CF improvements due to halving residential emissions to determine the energy and economic benefits (Text S2 and Figures S12 and S13). We find that the energy benefits from halving residential emissions are 10.3 and 2.5 TWh yr^–1^ over China and India, respectively. On the basis of 2020 electricity prices,^66^ this translates to economic benefits of US$878 million yr^–1^ and US$196 million yr^–1^, respectively. In comparison to the 2020 electricity generation of 260.5 and 60.4 TWh yr^–1^ from solar PV technology in China^67^ and India,^68^ respectively, these energy and economic benefits represent an approximately 4% improvement. Generally, regions where there are larger established PV installations will benefit more from stringent residential emission reductions. For example, four of the top five Chinese provinces or Indian states with the largest PV installations benefit the most from halving residential emissions. Even regions with moderate PV installations benefit from large CF improvements due to halving residential emissions: e.g., Henan province in China.

To show the benefits of reducing residential emissions more realistically, we do additional simulations in which we reduce residential emissions by 25%, 75%, and 100%. These simulations, together with the simulation of halving residential emissions, suggest that policies to reduce residential emissions will likely lead approximately linearly to improvements in PV efficiency and the associated rewards for the solar energy industry in East and South Asia (see approximately equal width of horizontal bars of different colors in Figure S12c,d and Figure S12e–h).

### Consistent Benefits throughout the Years

We provide the first quantitative assessment of the benefits to global and regional PV efficiency from halving air pollutant emissions in the anthropogenic source sectors. We present our results on decadal and corresponding seasonal scales because we find consistent benefits to PV efficiency throughout the years, particularly those from stringent residential emission reductions over Asia with respect to the proportion of occupied areas (Figure S14). The uncontrolled and inefficient combustion of solid fuels in residential devices is likely the prime culprit. This is supported by previous studies that show that completely removing residential emissions can achieve considerable air quality benefits,^69^ particularly in East^70,71^ and South Asia.^72^ Our work highlights that more realistic stringent reductions of residential emissions also lead to noticeable improvements in surface air quality with respect to PM_2.5_, i.e., PM with an aerodynamic diamater ⩽ 2.5 μm (Figure S15), which will benefit human health. The resulting improvements of PV efficiency will subsequently reduce the dependence on conventional energy generation, including the inefficient combustion of solid fuels over East and South Asia, which will further improve air quality, leading to a virtuous cycle.

### Role of Precipitation and Cleaning Panels

We follow ref (10) in using precipitation as the sole natural mechanism to reduce the impacts of PM soiling. We determine the influence of precipitation on reducing the impacts of PM soiling by comparing PV efficiency in model runs with and without the influence of precipitation (Figure S16). We find that precipitation plays an important role in shaping the spatial pattern of current-level PV efficiency, whose values would otherwise be reduced by more than 60% over resource-abundant regions, excluding Greenland and Antarctica.

On the basis of our analysis, halving anthropogenic PM emissions does not benefit desert regions where there are large PM soiling impacts on PV energy generation. These regions typically have a high abundance of natural dust (Figure S8) that are hardly removed by precipitation (Figure S9). A better strategy is routine sweeping of panels that overcomes the majority of PM soiling impacts (Figure S17). We find that even an annual sweeping routine will remove around 60% of PM soiling impacts in the Sahara, Arabian Peninsula, and Central Asia (Figure S18). A larger energy return will result from regularly sweeping at higher frequencies but will incur higher costs and higher risk of damaging PV panels. Clearly, further regional cost–benefit analyses are needed to balance the value of increased energy production versus costs associated with sweeping PV panels.

## Supporting Information Available

Additional information about the model design and evaluation, characteristics of the three panel settings, reference regions, distributions, interannual variabilities, and regional statistics, comparisons of PV efficiency, PM impacts, and benefits of reducing emissions, precipitation, and cleaning panels, cobenefits to the energy sector and surface air quality, and distributions of PM dry deposition fluxes and precipitation rates (PDF)
