# Supplementary material for: Source Sector Mitigation of Solar Energy Generation Losses Attributable to Particulate Matter Pollution
Source: Environ Sci Technol. 2022 Jun 1;56(12):8619–28. doi: 10.1021/acs.est.2c01175 (PMC9228073; doi:10.1021/acs.est.2c01175)
Supplement: Supplementary file 1 — es2c01175_si_001.pdf [file es2c01175_si_001.pdf]

Supporting Information for: Source Sector Mitigation of Solar Energy Generation Losses Attributable to Particulate Matter Pollution

Fei Yao[*,†] and Paul I. Palmer[†,‡]

†*School of GeoSciences, University of Edinburgh, Edinburgh, EH9 3FF, UK*

‡*National Centre for Earth Observation, University of Edinburgh, Edinburgh, EH9 3FF, UK*

E-mail: Fei.Yao@ed.ac.uk

Summary of contents: 33 pages, 2 texts, 18 figures, and 5 tables.

Text S1. Methods, results, implications, and limitations of model evaluation

We evaluate the integrated model from three perspectives by using a range of *in situ* observations. They are: 1) hourly global horizontal irradiance (GHI) observations from the Baseline Surface Radiation Network (BSRN)[1], which partly helps to evaluate real photovoltaic (PV) efficiency; 2) column aerosol optical depth (AOD) observations at every 15 minutes interval from the NASA AErosol RObotic NETwork (AERONET)[2], which partly helps to evaluate the impacts of atmospheric particulate matter (PM) on PV efficiency (dimming); and 3) hourly surface $PM_{2.5}$ (PM with aerodynamic diameter less than 2.5 μm) concentration observations falling within [0,3000] μg m^{-3} from the China National Environmental Monitoring Center (CNEMC) and OpenAQ, and its major chemical components observations mainly at monthly intervals from the literature[3], which partly helps to evaluate the impacts of deposited PM on PV efficiency (soiling).

To maximize the use of the available observation data, we sample model values with the locations and periods of observed values, and for GHI, AOD, and $PM_{2.5}$, we further compile the paired model and observed values into monthly composites to make the comparisons. We compare AODs at 550 nm, to which we interpolate AERONET AOD using a second-order polynomial fit of $\ln(AOD) \sim \ln(wavelength)$[4] that requires at least three data pairs encircling 550 nm. Model AODs at 550 nm are calculated based on dry mass concentrations of PM species and associated optical properties[5,6]. Model values of $PM_{2.5}$ and its major chemical components include the secondary aerosols, black carbon, accumulation mode sea salt, and dust of the first four size bins and 38% of the fifth size bin, and are calculated at a relative humidity of 35%, temperature of 298 K, and a pressure of 1013.25 hPa[7].

To describe the comparisons between model simulations and *in situ* observations we use the Pearson correlation coefficients, normalized mean bias $NMB = \frac{\sum_1^N (M-O)}{\sum_1^N O}100\%$, and normalized root mean square error $NRMSE = \frac{\sqrt{\frac{1}{N}\sum_1^N (M-O)^2}}{O_{max}-O_{min}}100\%$, where M and O denote

model and observed values, respectively, O_{max} and O_{min} denote the maximum and minimum observed values, respectively, and N denotes the number of comparison points. We report model performance statistics on both annual and seasonal scales to evaluate the integrated model capability during both periods of high and low solar insolation.

Figure S2 shows the site-scale evaluation of simulated GHI against BSRN GHI between 2008 and 2017. For Pearson correlation coefficients, approximately 98%, 93%, 93%, 94%, and 95% of the sites report statistically significant ($p < 0.05$) values, of which 100%, 98%, 91%, 97%, and 97% have values $\geq$ 0.7, during the entire, spring, summer, autumn, and winter periods, respectively. For NMB, approximately 94%, 98%, 87%, 85%, and 77% of the sites report values within $\pm$30% during the entire, spring, summer, autumn, and winter periods, respectively. For NRMSE, approximately 94%, 70%, 63%, 77%, and 69% of the sites report values no larger than 30% during the entire, spring, summer, autumn, and winter periods, respectively.

Figure S3 shows the site-scale evaluation of simulated AOD against AERONET AOD between 2008 and 2017. For Pearson correlation coefficients, approximately 70%, 54%, 50%, 51%, and 46% of the sites report statistically significant ($p < 0.05$) values, of which 55%, 66%, 63%, 73%, and 62% have values $\geq$ 0.7, during the entire, spring, summer, autumn, and winter periods, respectively. For NMB, approximately 84%, 86%, 82%, 80%, and 77% of the sites report values within $\pm$50% during the entire, spring, summer, autumn, and winter periods, respectively. For NRMSE, approximately 81%, 72%, 59%, 75%, and 63% of the sites report values no larger than 50% during the entire, spring, summer, autumn, and winter periods, respectively.

Figure S4 shows the site-scale evaluation of simulated $PM_{2.5}$ against CNEMC and OpenAQ $PM_{2.5}$ between 2008 and 2017. For Pearson correlation coefficients, approximately 86%, 19%, 40%, 35%, and 52% of the sites report statistically significant ($p < 0.05$) values, of which 51%, 46%, 55%, 58%, and 70% have values $\geq$ 0.7, during the entire, spring, summer, autumn, and winter periods, respectively. For NMB, approximately 70%, 54%, 55%,

62%, and 79% of the sites report values within ±50% during the entire, spring, summer, autumn, and winter periods, respectively. For NRMSE, approximately 87%, 33%, 33%, 53%, and 60% of the sites report values no larger than 50% during the entire, spring, summer, autumn, and winter periods, respectively.

Figure S5 shows the evaluation of simulated $PM_{2.5}$ chemical composition concentrations against ground observation data collected from the literature[3]. On the annual scale, the Pearson correlation coefficients, NMB, and NRMSE between model and observed values range from 0.42 to 0.59, from -29% to 14%, and from 9% to 16% for different species, respectively. Admittedly, the Pearson correlation coefficients are not particularly high but most of them are statistically significant ($p < 0.05$) and comparable to those of similar one-way models[8], albeit slightly smaller than those of two-way coupled models[3]. On the seasonal scale, both superior and inferior statistics exist, reflecting the possible varying model capability in different seasons but note that the decreasing number of observations may also result in some fluctuations.

The above statistics suggest that the integrated model has generally reproduced the observed variations in GHI, AOD, and $PM_{2.5}$ and its major chemical composition concentrations during both periods of high and low solar insolation. In particular, the integrated model has the best performance for GHI, followed by AOD then $PM_{2.5}$ and its chemical composition concentrations. This may suggest the higher accuracy in real PV efficiency than PM dimming and soiling impacts. Uncertainties in the bottom-up inventories and the underlying physical and chemical processes could be the plausible explanation, but arguably, this is somewhat ubiquitous in most process-based models.

We highlight three points associated with our model evaluation. First, the model evaluation includes unavoidable discrepancies in temporal and spatial resolutions between simulations and observations. Second, although each source of observations is temporally and spatially incomplete, it could be argued collectively that the various sources of observations supplement each other to evaluate the integrated model. Third, $PM_{2.5}$ and its major chemi-

cal components exclude those particles with an aerodynamic diameter above 2.5 μm, so may not be the most appropriate metric to evaluate the integrated model. But they appear to be the most available metric given that our immediate focus in this work is anthropogenic sources of PM which mainly contribute to $PM_{2.5}$, and that the well-documented harmful impacts of $PM_{2.5}$ and its major chemical components on human health lead to their abundant data sources.

Text S2. Calculations of energy and economic benefits

The installed PV capacities as of 2019 we collected from Chinese[9] and Indian[10] national energy-related administration are on the province/state level and have a distinction between distributed and utility-scale PV installations. Therefore, we follow ref 11 to assume Tilt and OAT panels for distributed and utility-scale PV installations, respectively. For each province/state, we extract capacity factor improvements (ΔCFs) in grid cells within that province/state. For grid cells overlapping more than one province/state, we split them along provincial/state boundaries with a geographical information system program. Further using areas of these (split) grid cells, we derive the provincial/state-wise area-weighted mean ΔCFs. Energy benefits (potential increases in PV electricity generation) are then computed on the provincial/state level by multiplying ΔCFs for Tilt and OAT with one year length and the installed capacities of distributed and utility-scale PV, respectively. Economic benefits are further derived by multiplying these energy benefits with the national mean electricity price for households in China ($\approx$ US\$0.085 kWh^{-1}) and India ($\approx$ US\$0.079 kWh^{-1}) in 2020, respectively[12].

Figures S1–S18

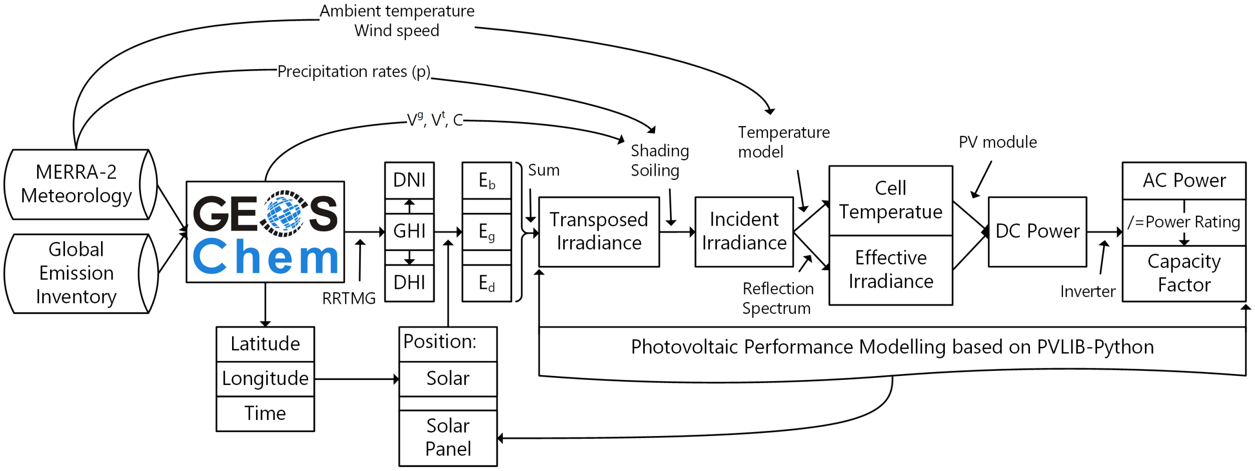

Figure S1. Workflow to estimate solar photovoltaic electricity generation efficiency in this study. Full definitions for abbreviations include: 1) global horizontal irradiance (GHI) simulated from GEOS-Chem coupled with rapid radiative transfer model for GCMs (RRTMG), and it is subsequently decomposed to direct normal irradiance (DNI) and diffuse horizontal irradiance (DHI); 2) beam (E_b), ground-reflected (E_g), and sky-diffuse (E_d) components of transposed irradiance; 3) PM gravitational (V^g) and turbulent (V^t) velocities; 4) PM dry mass concentrations (C); 5) direct (DC) and alternating (AC) current powers.

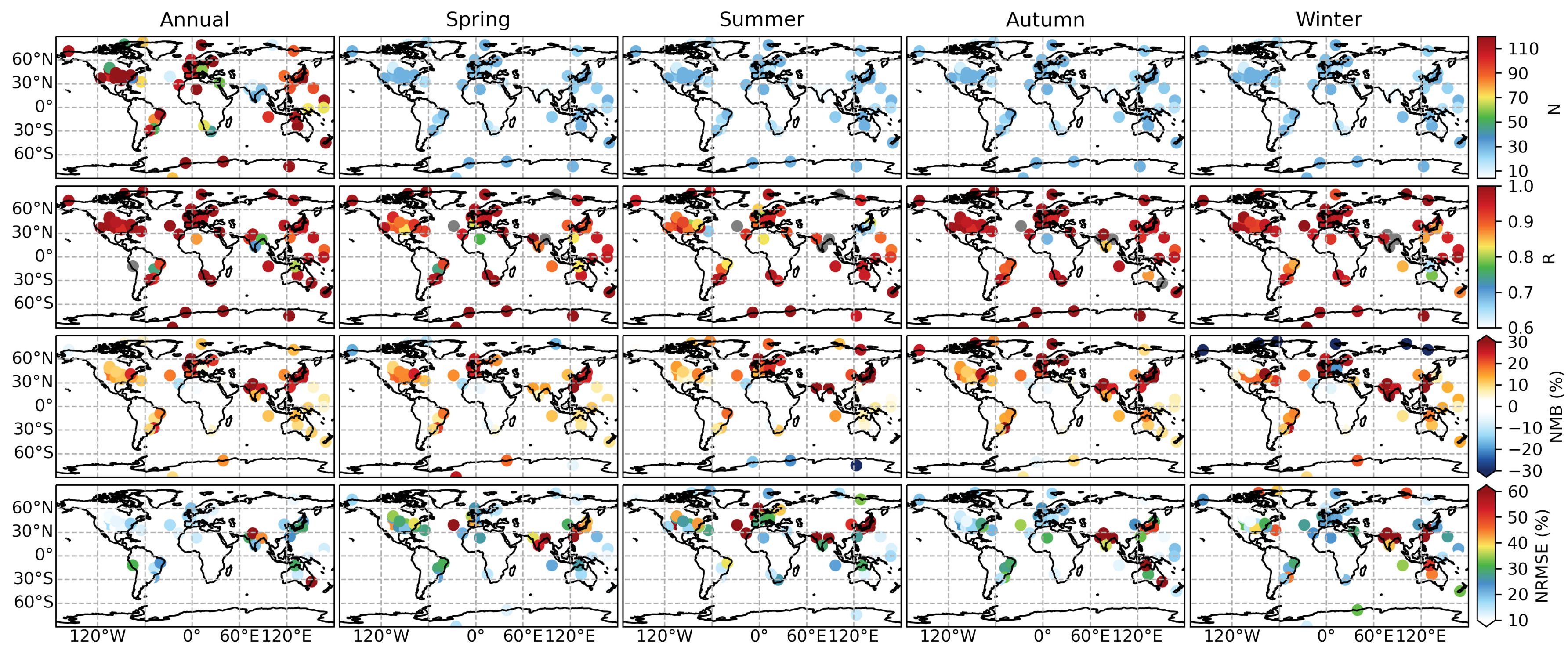

Figure S2. Site-scale evaluation of simulated GHI against BSRN GHI observations between 2008 and 2017. Pearson correlation coefficients with $p \geq 0.05$ are marked as grey dots.

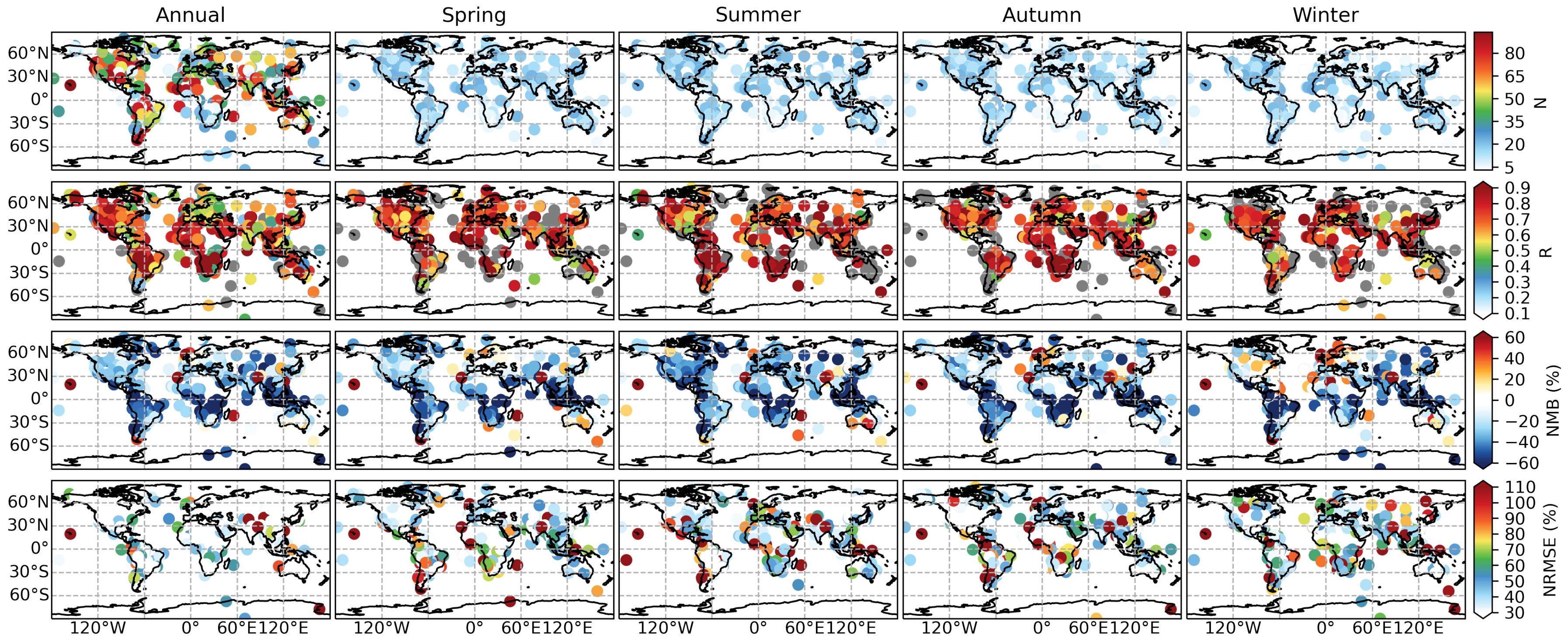

Figure S3. Site-scale evaluation of simulated AOD against AERONET AOD observations between 2008 and 2017. Pearson correlation coefficients with $p \geq 0.05$ are marked as grey dots.

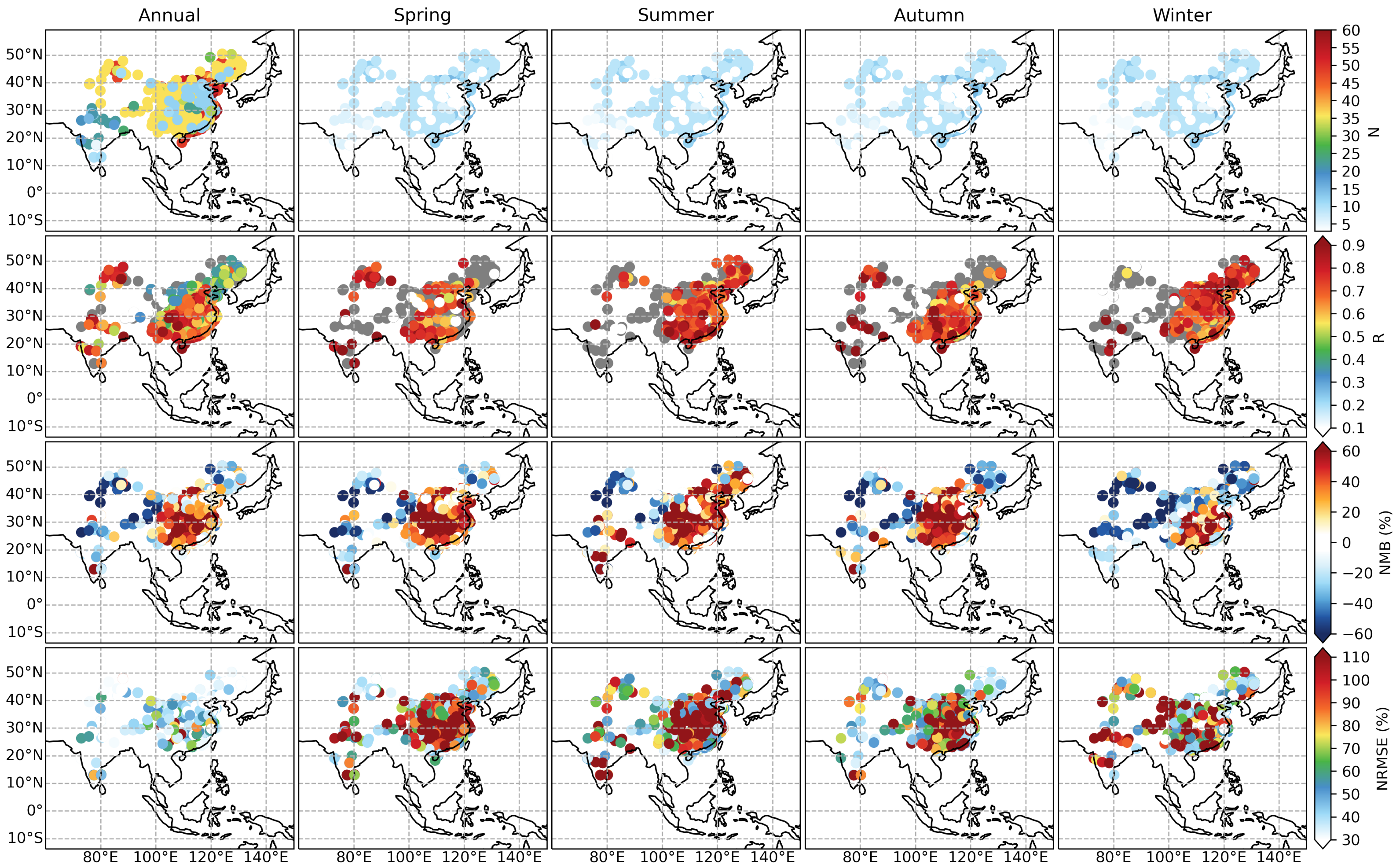

Figure S4. Site-scale evaluation of simulated $PM_{2.5}$ against CNEMC and OpenAQ $PM_{2.5}$ observations between 2008 and 2017. Pearson correlation coefficients with $p \geq 0.05$ are marked as grey dots.

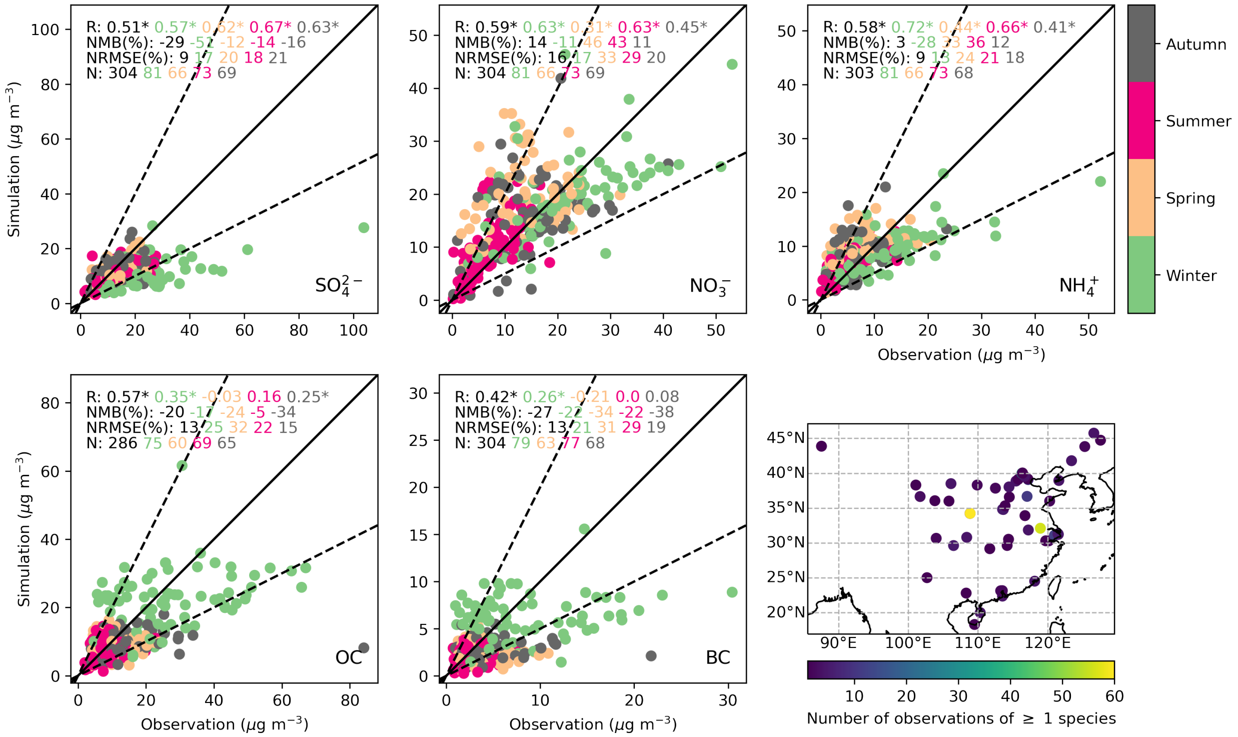

Figure S5. Evaluation of simulated $PM_{2.5}$ chemical composition concentrations against ground-based observations collected from the literature[3]. The solid line corresponds to the 1:1 line, and the dashed lines correspond to the 1:2 and 2:1 lines. Statistically significant ($p < 0.05$) Pearson correlation coefficients are marked with a $*$. The bottom right subplot shows the number of observations of at least one $PM_{2.5}$ chemical component over each monitoring location.

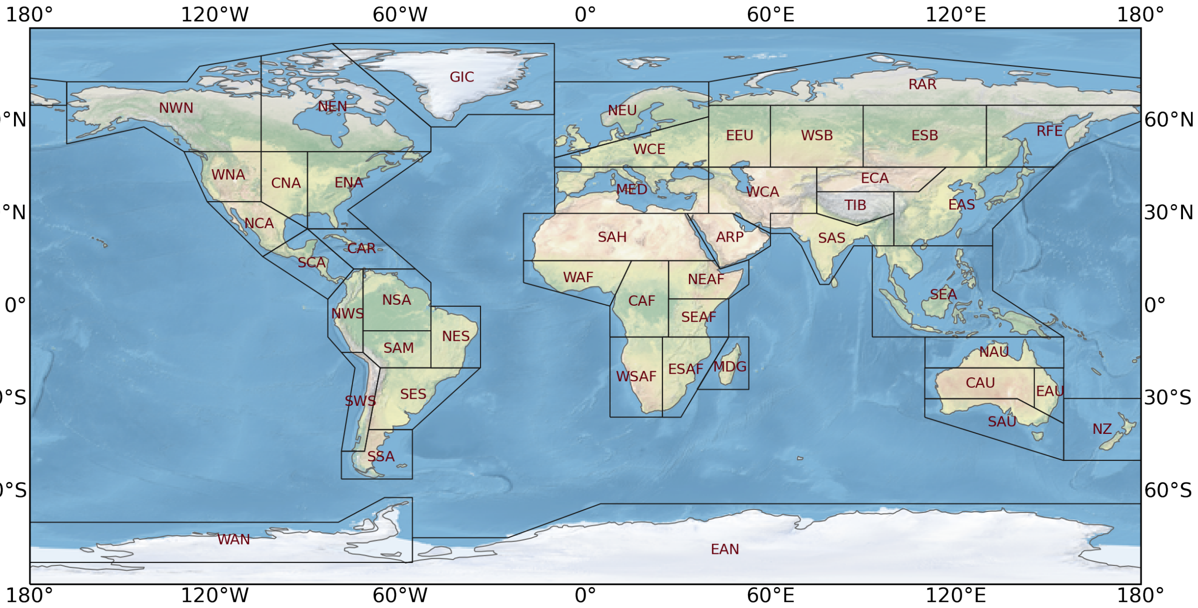

0	GIC	Greenland/Iceland	23	NEAF	N.Eastern-Africa
1	NWN	N.W.North-America	24	SEAF	S.Eastern-Africa
2	NEN	N.E.North-America	25	WSAF	W.Southern-Africa
3	WNA	W.North-America	26	ESAF	E.Southern-Africa
4	CNA	C.North-America	27	MDG	Madagascar
5	ENA	E.North-America	28	RAR	Russian-Arctic
6	NCA	N.Central-America	29	WSB	W.Siberia
7	SCA	S.Central-America	30	ESB	E.Siberia
8	CAR	Caribbean	31	RFE	Russian-Far-East
9	NWS	N.W.South-America	32	WCA	W.C.Asia
10	NSA	N.South-America	33	ECA	E.C.Asia
11	NES	N.E.South-America	34	TIB	Tibetan-Plateau
12	SAM	South-American-Monsoon	35	EAS	E.Asia
13	SWS	S.W.South-America	36	ARP	Arabian-Peninsula
14	SES	S.E.South-America	37	SAS	S.Asia
15	SSA	S.South-America	38	SEA	S.E.Asia
16	NEU	N.Europe	39	NAU	N.Australia
17	WCE	West&Central-Europe	40	CAU	C.Australia
18	EEU	E.Europe	41	EAU	E.Australia
19	MED	Mediterranean	42	SAU	S.Australia
20	SAH	Sahara	43	NZ	New-Zealand
21	WAF	Western-Africa	44	EAN	E.Antarctica
22	CAF	Central-Africa	45	WAN	W.Antarctica

Figure S6. Definitions of 46 AR6 land regions[13] used in this study to calculate regional area-weighted mean variables.

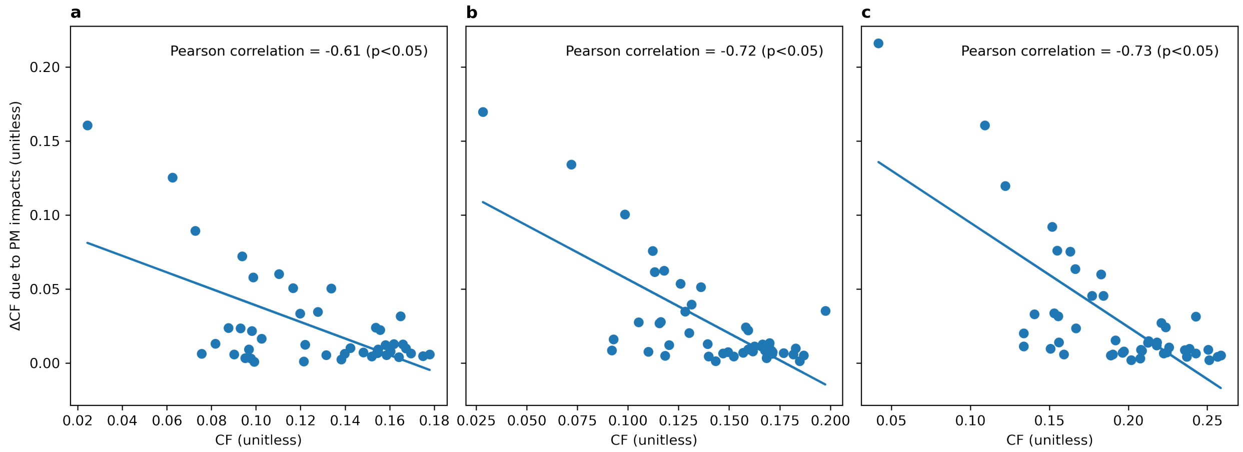

Figure S7. Comparison of regional area-weighted averages of decadal mean (2008–2017) PV efficiency and PM impacts for (a) flat, (b) tilt, and (c) one-axis tracking panels. Please refer to Figure S6 for definitions of regions that are used here for taking regional area-weighted averages.

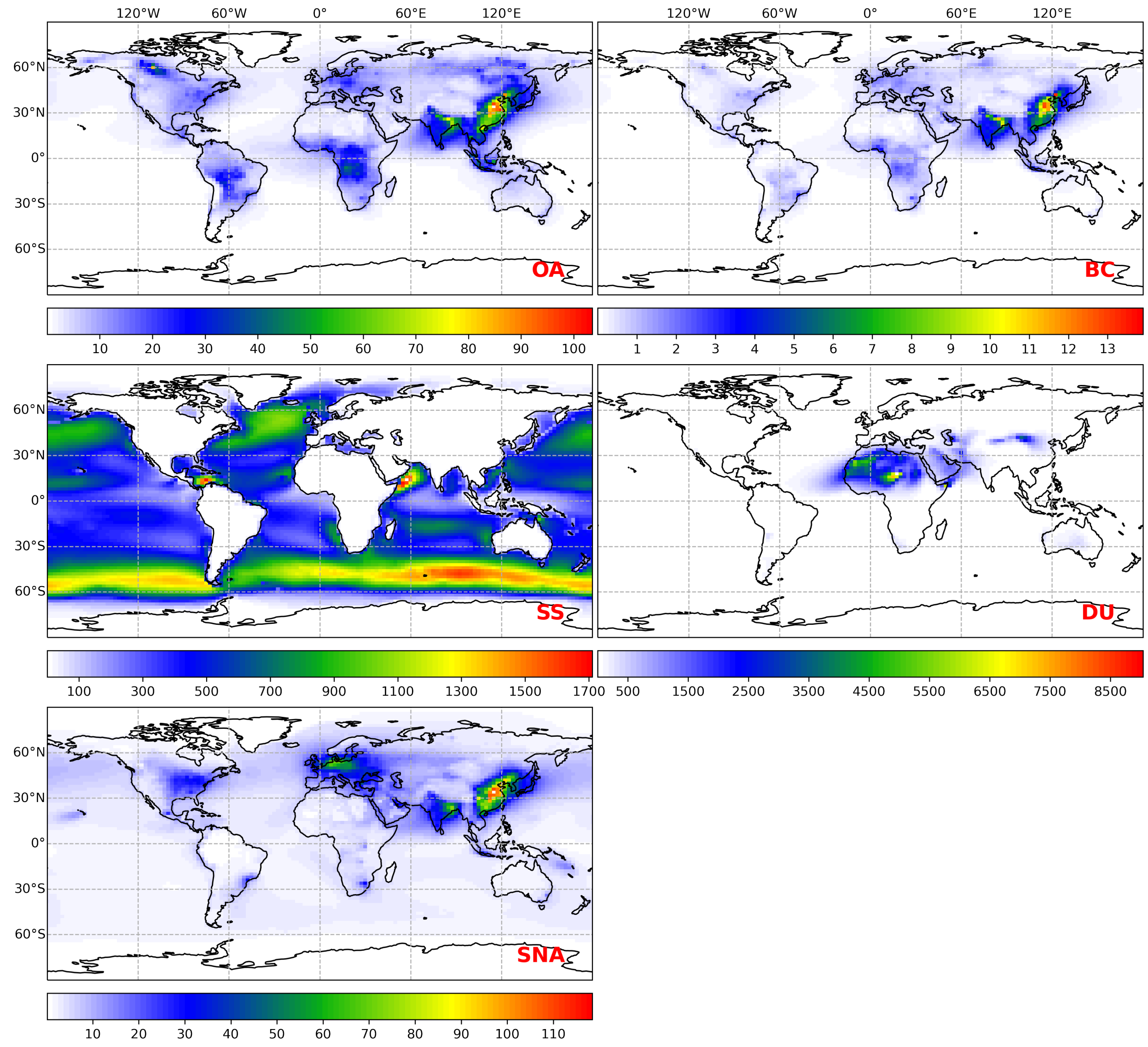

Figure S8. Geographical distributions of decadal mean (2008–2017) deposition fluxes ($\mu g/m^2/hr$) for each PM species: organic aerosol (OA), black carbon (BC), sea salt (SS), dust (DU), and sulfate–nitrate–ammonium (SNA) at 2° (latitude) × 2.5° (longitude) resolution.

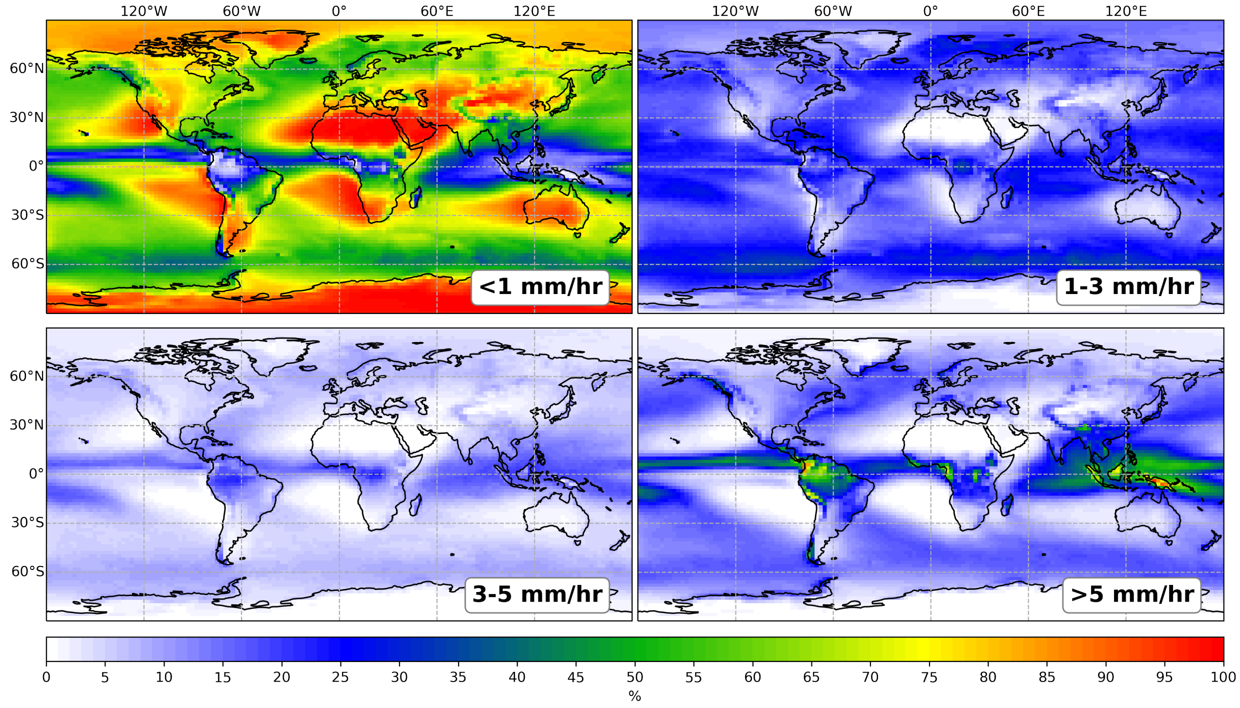

Figure S9. Frequency of precipitation rates of < 1 mm/hr, 1–3 mm/hr, 3–5 mm/hr, and > 5 mm/hr from MERRA-2 between 2008 and 2017.

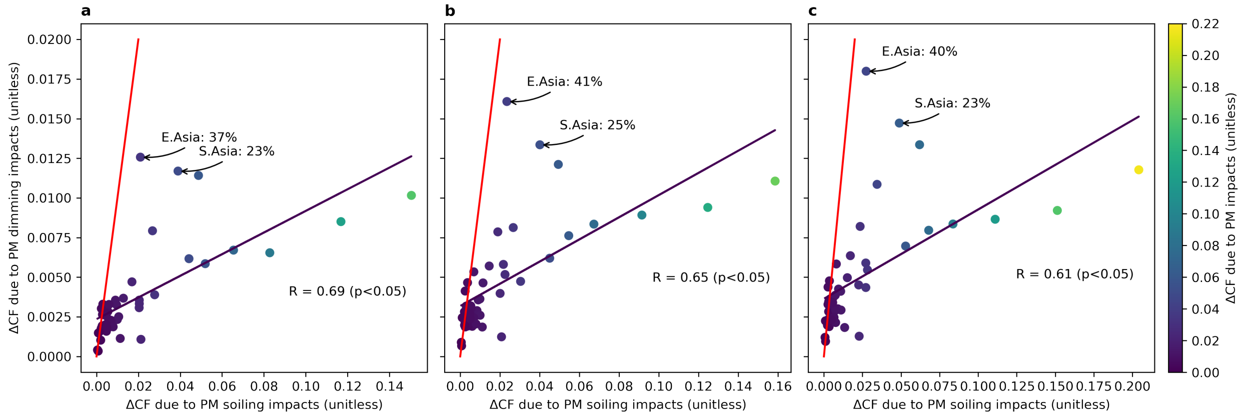

Figure S10. Comparison of regional area-weighted averages of decadal mean (2008–2017) PM dimming and soiling impacts for (a) flat, (b) tilt, and (c) one-axis tracking panels. The blue lines are the regression lines between PM dimming and soiling impacts, with R denoting the Pearson correlation coefficients. The red lines are the 1:1 lines. The vertical colorbar represents PM total impacts. The percentages following E.Asia and S.Asia where there are the largest PM dimming are the shares of PM dimming over these two regions. Please refer to Figure S6 for definitions of regions that are used here for taking regional area-weighted averages.

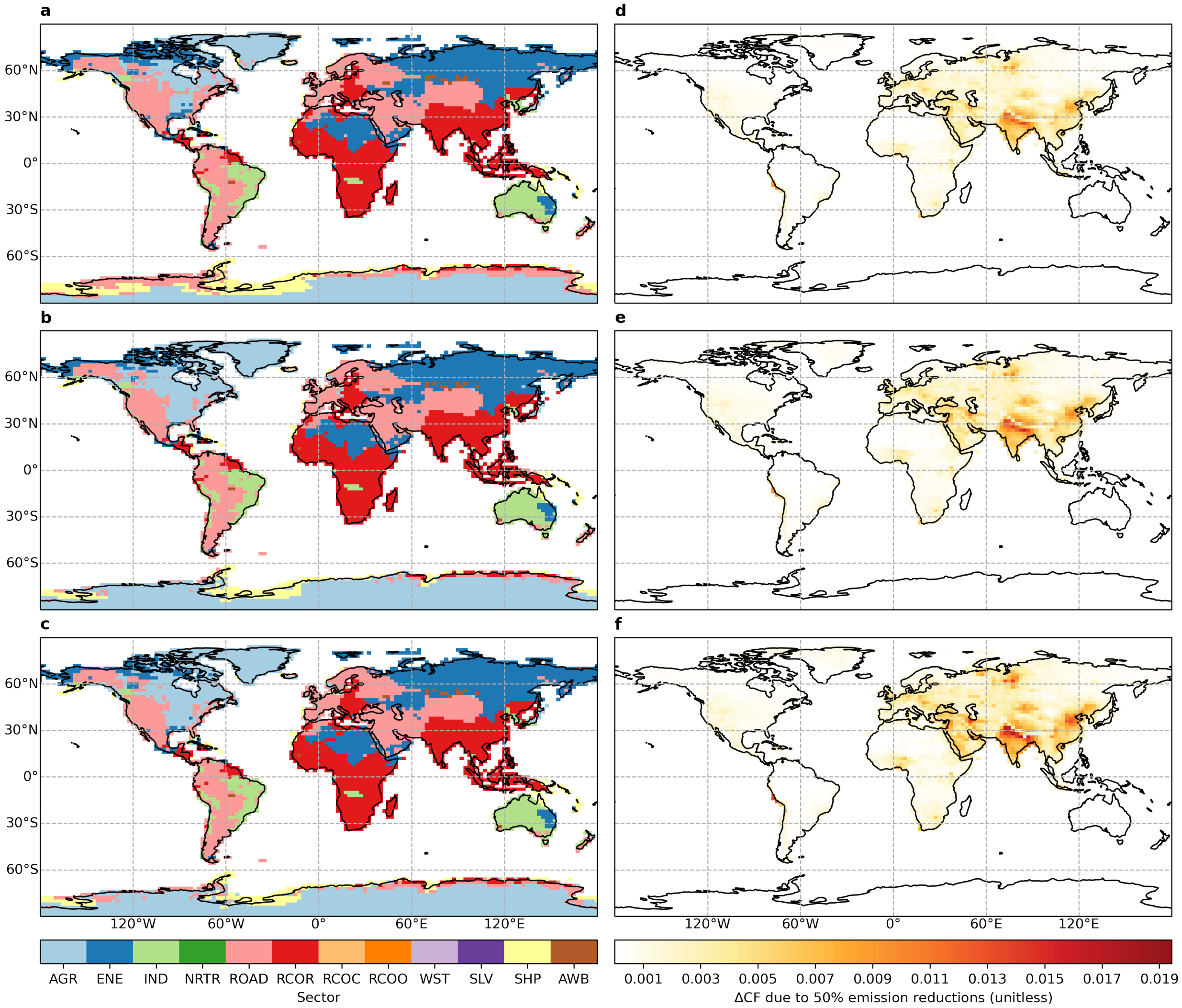

Figure S11. Geographical distributions of (a-c) source sectors from which halving emissions provides (d-f) maximum decadal mean (2008–2017) total benefits for (a, d) flat, (b, e) tilt, and (c, f) one-axis tracking panels. Full definitions for source sectors are noncombustion agriculture (AGR), energy generation (ENE), industrial processes (IND), nonroad (NRTR) and on-road (ROAD) transportation, separate residential (RCOR), commercial (RCOC), and other (RCOO) sectors, waste (WST), solvent use (SLV), international shipping (SHP), and agricultural waste burning (AWB).

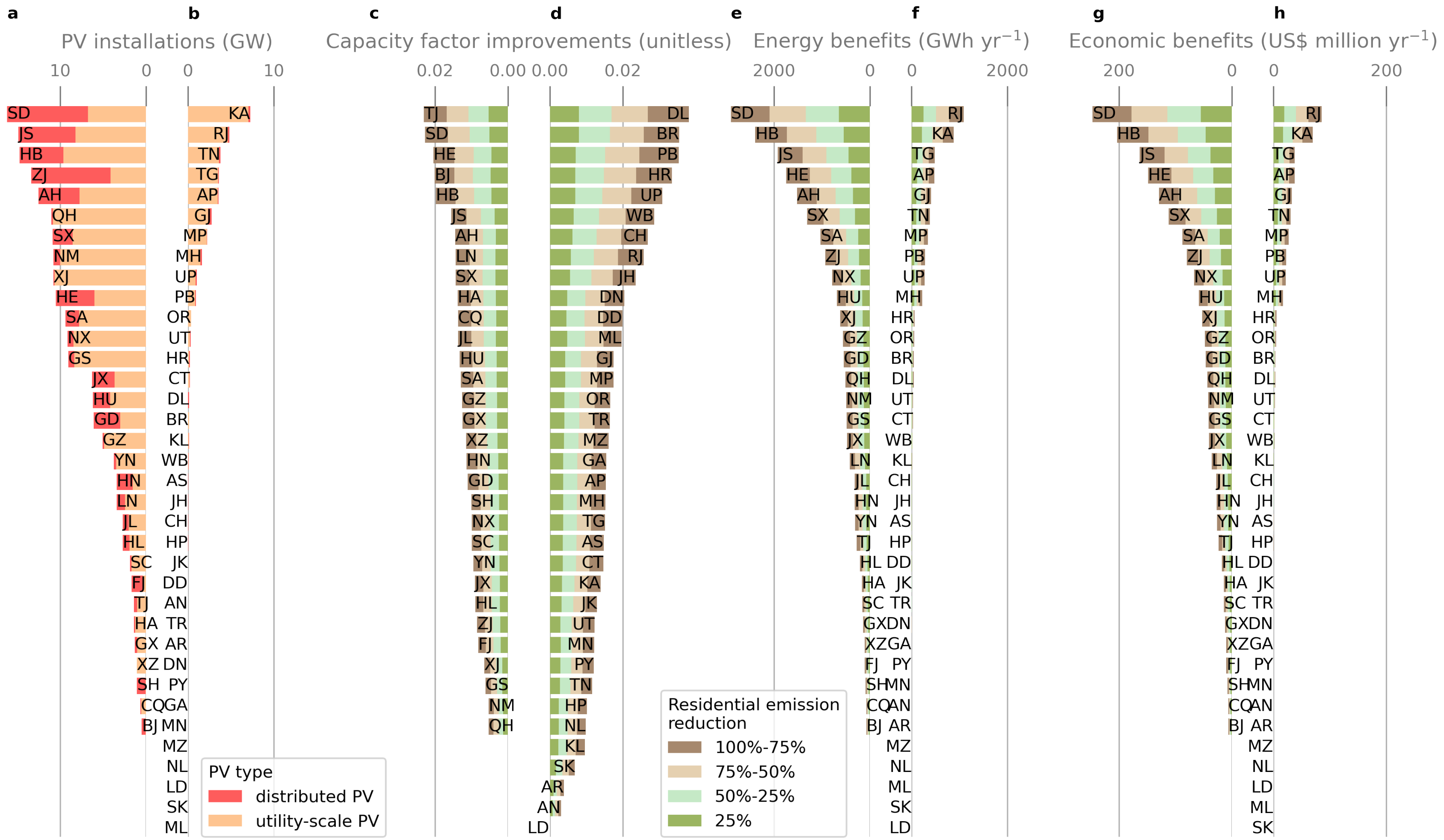

Figure S12. Potential additional (e, f) energy (GWh yr^{-1}) and (g, h) economic (US$ million yr^{-1}) benefits by combining (c, d) decadal mean (2008–2017) capacity factor improvements (OAT panels as an illustration) from reducing residential emissions with (a, b) the installed capacities as of 2019 in (a, c, e, g) China and (b, d, f, h) India. Please refer to Figure S13 for definitions of Chinese provinces and Indian states that are presented here.

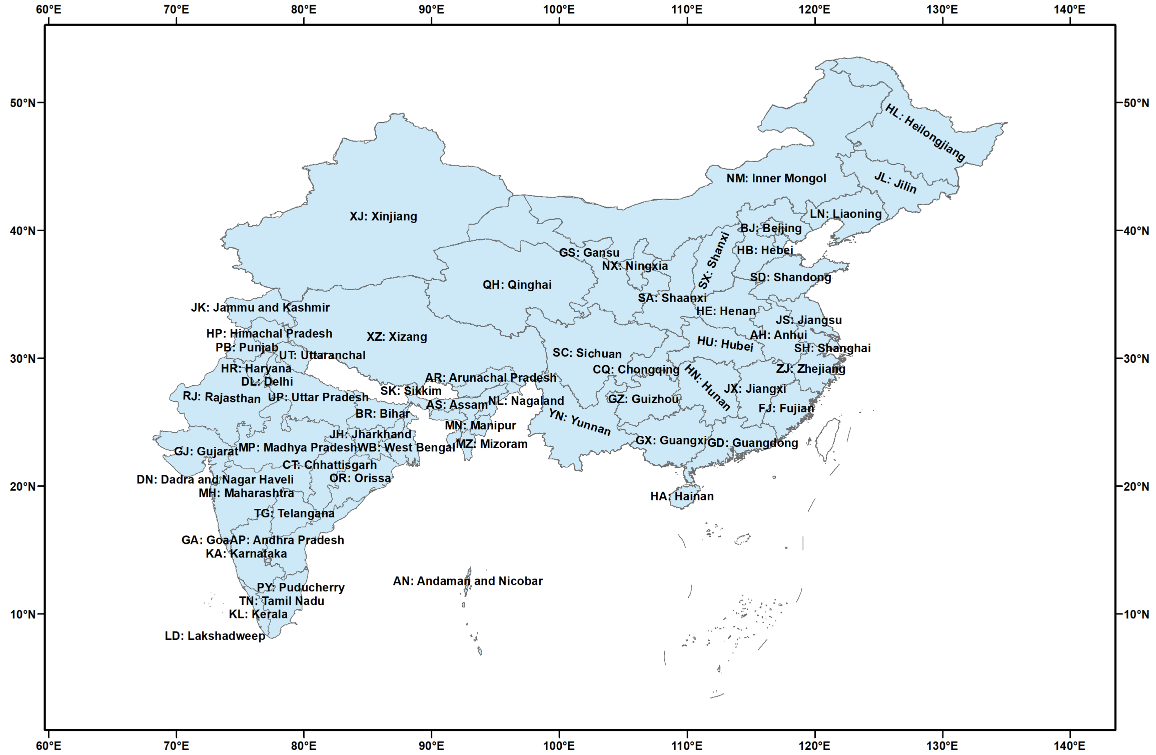

Figure S13. Definitions of Chinese provinces and Indian states used in Figure S12.

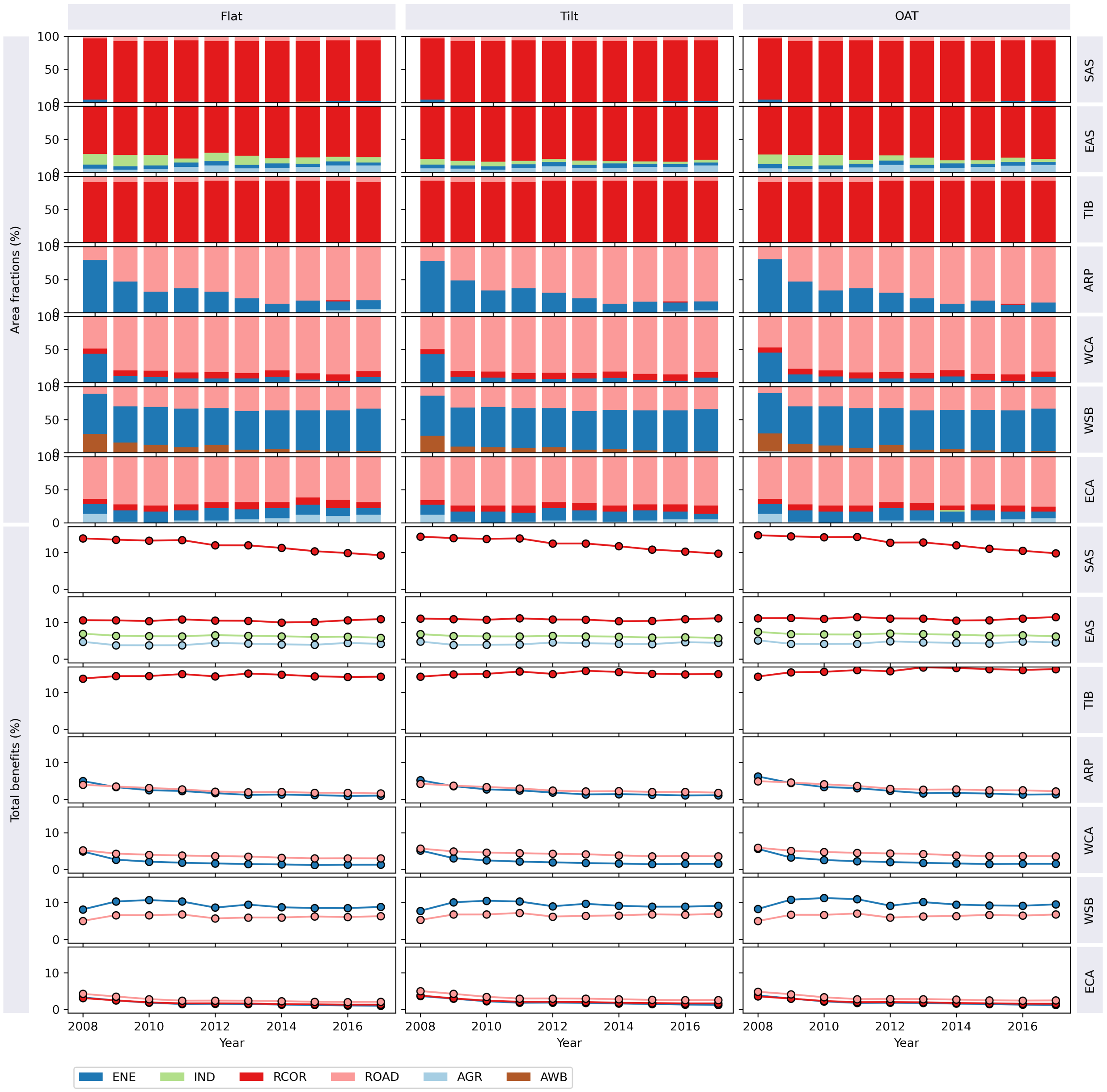

Figure S14. Consistent benefits to PV efficiency throughout years particularly those from stringent residential emission reductions over Asia with respect to the proportion of occupied areas. Please refer to Figure S6 for definitions of regions that are presented here. Full definitions for source sectors are noncombustion agriculture (AGR), energy generation (ENE), industrial processes (IND), nonroad (NRTR) and on-road (ROAD) transportation, separate residential (RCOR), commercial (RCOC), and other (RCOO) sectors, waste (WST), solvent use (SLV), international shipping (SHP), and agricultural waste burning (AWB).

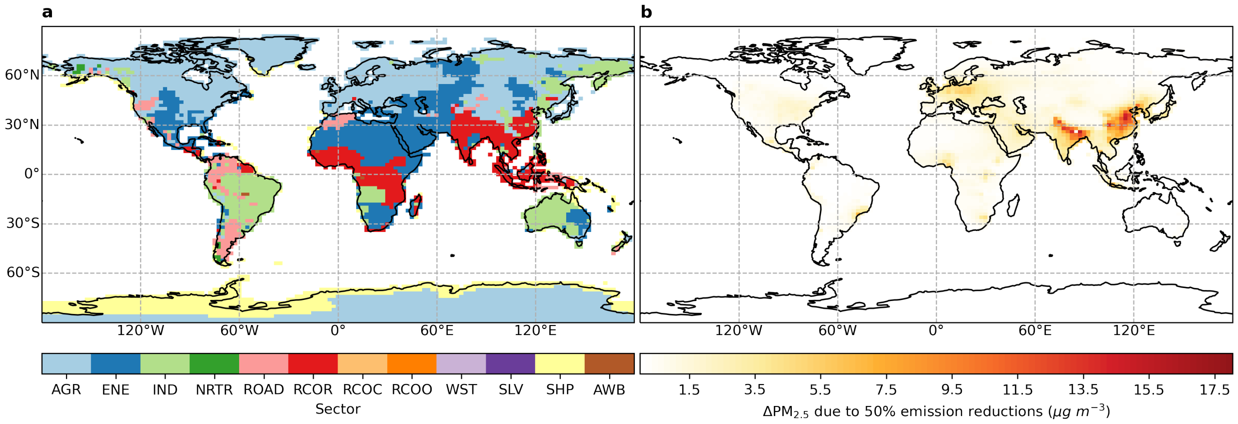

Figure S15. Geographical distributions of (a) sectors from which halving emissions provides (b) maximum decadal mean (2008–2017) cobenefits to surface air quality with respect to PM with an aerodynamic diameter $\leq$ 2.5 μm. Full definitions for source sectors are noncombustion agriculture (AGR), energy generation (ENE), industrial processes (IND), nonroad (NRTR) and on-road (ROAD) transportation, separate residential (RCOR), commercial (RCOC), and other (RCOO) sectors, waste (WST), solvent use (SLV), international shipping (SHP), and agricultural waste burning (AWB).

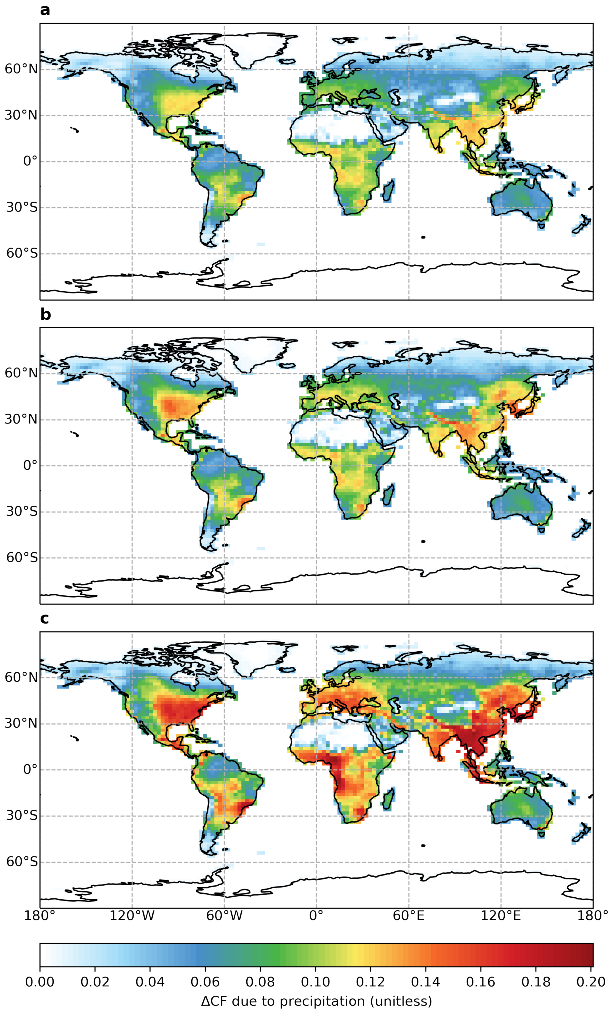

Figure S16. Geographical distributions of decadal mean (2008–2017) cleaning benefits resulting from precipitation for (a) flat, (b) tilt, and (c) one-axis tracking panels.

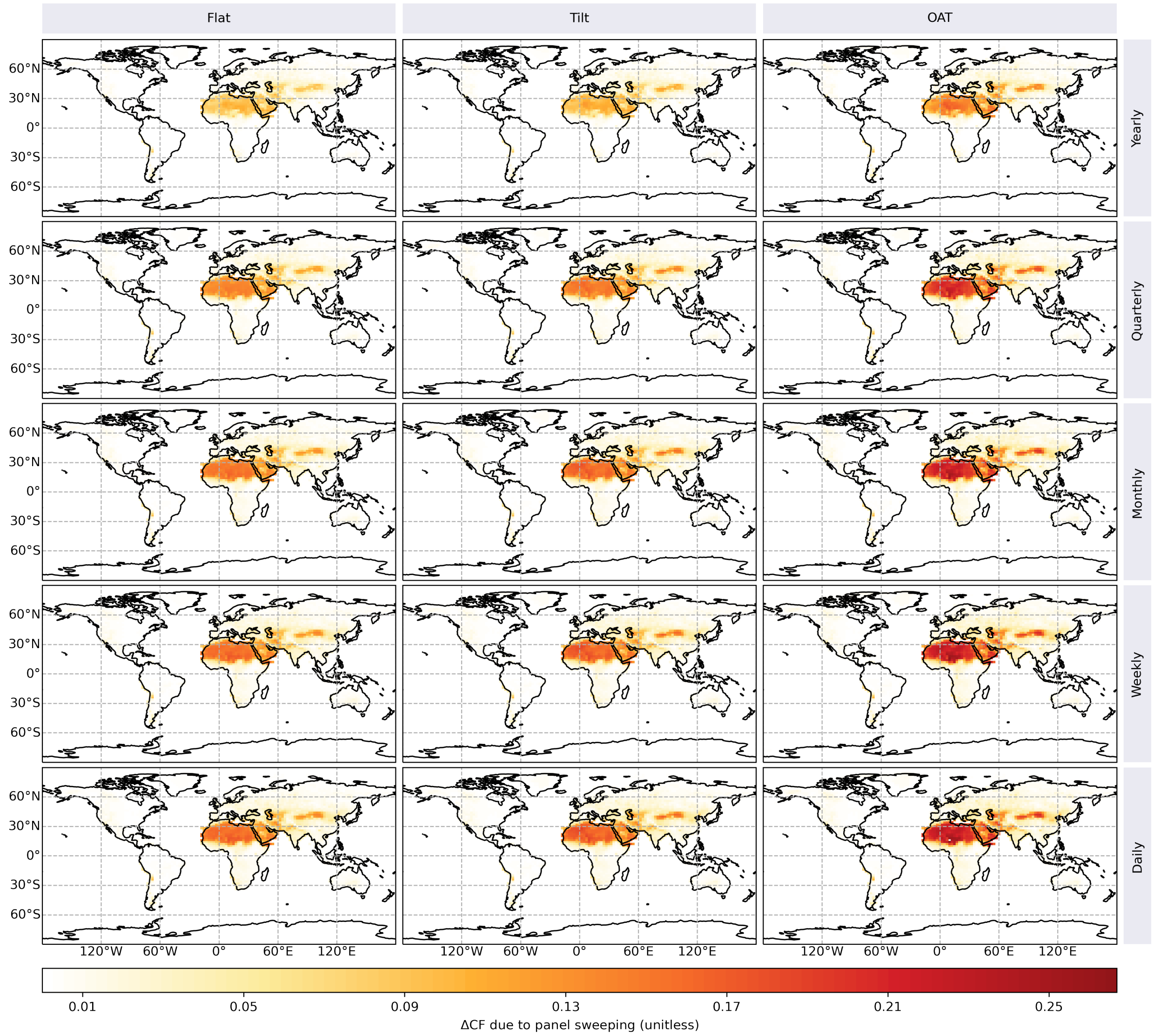

Figure S17. Geographical distributions of decadal mean (2008–2017) cleaning benefits resulting from various frequencies of panel sweeping for flat, tilt, and one-axis tracking panels.

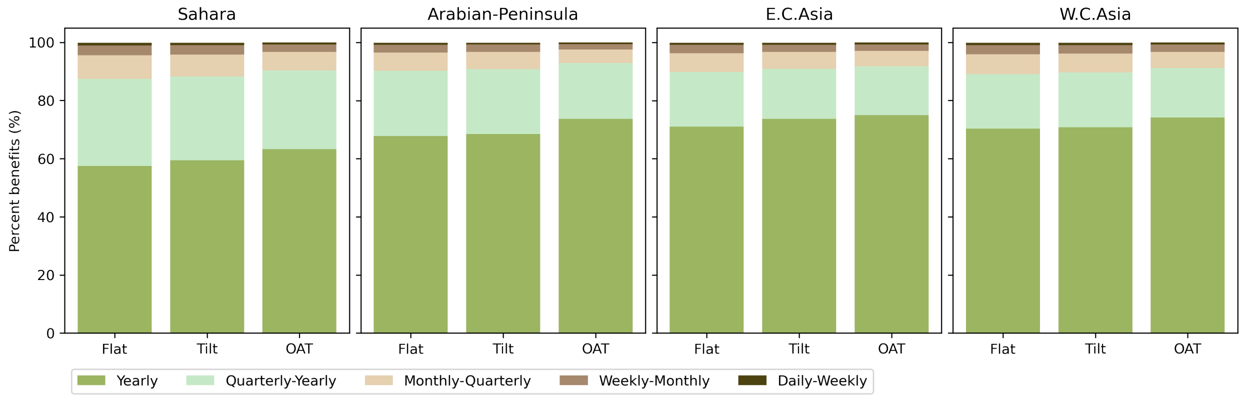

Figure S18. Regional area-weighted averages of decadal mean (2008–2017) percent cleaning benefits (relative to PM soiling impacts) resulting from various frequencies of panel sweeping, in several regions of interest. Please refer to Figure S6 for definitions of regions that are presented here.

Tables S1–S5

Table S1. Solar panel settings investigated in this study.

Name	Abbreviation	Descriptions
Horizontal fixed panels	Flat	Panels are fix mounted and horizontal
Optimally tilted panels	Tilt	Panels are fixed mounted, tilted at the latitude angle, and oriented to the equator
One-axis tracking panels	OAT	Panels rotate around one axis from east to west to track the sun throughout the day

Table S2. Measured optical properties of deposited PM taken from refs 14 and 15 used in this study.

Species	E_{abs} (m^2g^{-1})	β	E_{scat} (m^2g^{-1})
Dust	0.02	0.02	1.00
Organic carbon	0.00	0.30	4.00
Black carbon	8.00	0.30	0.00
Sulfate-nitrate-ammonium	0.00	0.30	4.00

Table S3. The proportion of occupied areas, and regional area-weighted mean brightening benefits and PM dimming impacts and their ratios of sectors from which halving emissions provides the largest decadal and corresponding seasonal mean brightening benefits, in various regions of interest. For brevity, we sort sectors in descending order by their mean proportions of occupied areas of the three panels, and only keep to maximum two sectors whose cumulative mean proportions $\geq$ 75%. Please refer to Fig. S6 for definitions of regions that are presented here. Full definitions for source sectors are noncombustion agriculture (AGR), energy generation (ENE), industrial processes (IND), nonroad (NRTR) and on-road (ROAD) transportation, separate residential (RCOR), commercial (RCOC), and other (RCOO) sectors, waste (WST), solvent use (SLV), international shipping (SHP), and agricultural waster burning (AWB).

Period	Region	Sector	Panel setting	Area fractions (%)	Brightening benefits (unitless)	PM dimming impacts (unitless)	Brightening benefits (%)
Annual	E.Asia	RCOR	Flat	46	0.0010	0.0126	8
			Tilt	58	0.0015	0.0161	9
			OAT	50	0.0016	0.0180	9
		AGR	Flat	43	0.0010	0.0126	8
			Tilt	37	0.0013	0.0161	8
			OAT	40	0.0015	0.0180	8
	S.Asia	RCOR	Flat	93	0.0014	0.0117	12
			Tilt	93	0.0017	0.0134	12
			OAT	93	0.0018	0.0147	12
	West&Central-Europe	AGR	Flat	100	0.0006	0.0047	13
			Tilt	100	0.0011	0.0079	13
			OAT	100	0.0011	0.0082	13
DJF	E.Asia	RCOR	Flat	99	0.0017	0.0118	15
			Tilt	99	0.0031	0.0216	14
			OAT	99	0.0028	0.0185	15
	S.Asia	RCOR	Flat	94	0.0018	0.0115	15
			Tilt	94	0.0026	0.0168	16
			OAT	94	0.0024	0.0152	16
	West&Central-Europe	AGR	Flat	100	0.0003	0.0023	12
			Tilt	100	0.0011	0.0085	13
			OAT	100	0.0007	0.0055	13

Table S3 Continued.

Period	Region	Sector	Panel setting	Area fractions (%)	Brightening benefits (unitless)	PM dimming impacts (unitless)	Brightening benefits (%)
MAM	E.Asia	AGR	Flat	79	0.0016	0.0169	10
			Tilt	80	0.0017	0.0178	10
			OAT	80	0.0024	0.0228	10
	S.Asia	RCOR	Flat	82	0.0015	0.0146	10
			Tilt	82	0.0016	0.0147	11
			OAT	81	0.0019	0.0178	11
	West&Central-Europe	AGR	Flat	100	0.0010	0.0072	13
			Tilt	100	0.0013	0.0094	14
			OAT	100	0.0016	0.0115	14
JJA	E.Asia	IND	Flat	69	0.0012	0.0113	10
			Tilt	68	0.0010	0.0096	10
			OAT	72	0.0017	0.0163	11
		RCOR	Flat	14	0.0007	0.0113	6
			Tilt	14	0.0006	0.0096	6
			OAT	13	0.0010	0.0163	6
	S.Asia	RCOR	Flat	66	0.0008	0.0107	8
			Tilt	66	0.0007	0.0093	8
			OAT	65	0.0011	0.0144	8
		ENE	Flat	29	0.0007	0.0107	6
			Tilt	29	0.0006	0.0093	6
			OAT	29	0.0009	0.0144	6
	West&Central-Europe	AGR	Flat	67	0.0007	0.0058	12
			Tilt	67	0.0007	0.0055	12
			OAT	67	0.0011	0.0092	12
		ENE	Flat	33	0.0004	0.0058	7
			Tilt	33	0.0004	0.0055	7
			OAT	33	0.0007	0.0092	8
SON	E.Asia	AGR	Flat	56	0.0010	0.0103	9
			Tilt	55	0.0014	0.0153	9
			OAT	55	0.0014	0.0142	10
		IND	Flat	38	0.0009	0.0103	9
			Tilt	38	0.0012	0.0153	8
			OAT	39	0.0013	0.0142	9
	S.Asia	RCOR	Flat	92	0.0013	0.0099	13
			Tilt	93	0.0017	0.0127	14
			OAT	91	0.0016	0.0116	14
	West&Central-Europe	AGR	Flat	100	0.0005	0.0035	15
			Tilt	100	0.0012	0.0081	15
			OAT	100	0.0010	0.0065	15

Table S4. The proportion of occupied areas, and regional area-weighted mean cleaning benefits and PM soiling impacts and their ratios of sectors from which halving emissions provides the largest decadal and corresponding seasonal mean cleaning benefits, in various regions of interest. For brevity, we sort sectors in descending order by their mean proportions of occupied areas of the three panels, and only keep to maximum two sectors whose cumulative mean proportions $\geq 75\%$. Please refer to Fig. S6 for definitions of regions that are presented here. Full definitions for source sectors are noncombustion agriculture (AGR), energy generation (ENE), industrial processes (IND), nonroad (NRTR) and on-road (ROAD) transportation, separate residential (RCOR), commercial (RCOC), and other (RCOO) sectors, waste (WST), solvent use (SLV), international shipping (SHP), and agricultural waster burning (AWB).

Period	Region	Sector	Panel setting	Area fractions (%)	Cleaning benefits (unitless)	PM soiling impacts (unitless)	Cleaning benefits (%)
Annual	S.Asia	RCOR	Flat	93	0.0045	0.0389	12
			Tilt	93	0.0048	0.0401	12
			OAT	93	0.0061	0.0489	12
	Tibetan-Plateau	RCOR	Flat	91	0.0042	0.0278	15
			Tilt	91	0.0048	0.0304	16
			OAT	91	0.0046	0.0272	17
	Arabian-Peninsula	ROAD	Flat	78	0.0023	0.1168	2
			Tilt	78	0.0028	0.1247	2
			OAT	78	0.0043	0.1513	3
	W.C.Asia	ROAD	Flat	87	0.0021	0.0655	3
			Tilt	87	0.0026	0.0673	4
			OAT	87	0.0033	0.0837	4
	E.Asia	RCOR	Flat	90	0.0025	0.0210	12
			Tilt	90	0.0028	0.0235	12
			OAT	89	0.0034	0.0274	13
	W.Siberia	ENE	Flat	52	0.0019	0.0204	10
			Tilt	52	0.0023	0.0226	10
			OAT	52	0.0029	0.0282	10
		ROAD	Flat	37	0.0013	0.0204	7
			Tilt	37	0.0016	0.0226	7
			OAT	37	0.0020	0.0282	7
	E.C.Asia	ROAD	Flat	67	0.0019	0.0828	2
			Tilt	72	0.0025	0.0915	3
			OAT	74	0.0030	0.1110	3
		ENE	Flat	14	0.0010	0.0828	1
			Tilt	15	0.0014	0.0915	2
			OAT	14	0.0017	0.1110	2
DJF	S.Asia	RCOR	Flat	93	0.0047	0.0365	13
			Tilt	93	0.0058	0.0450	13
			OAT	93	0.0068	0.0476	14
	Tibetan-Plateau	RCOR	Flat	91	0.0032	0.0202	16
			Tilt	91	0.0049	0.0297	17
			OAT	91	0.0038	0.0218	18
	Arabian-Peninsula	ROAD	Flat	71	0.0019	0.0948	2
			Tilt	71	0.0029	0.1273	2
			OAT	71	0.0039	0.1359	3
		ENE	Flat	27	0.0013	0.0948	1
			Tilt	27	0.0020	0.1273	2
			OAT	29	0.0027	0.1359	2
	W.C.Asia	ROAD	Flat	85	0.0013	0.0397	3
			Tilt	86	0.0023	0.0585	4
			OAT	85	0.0021	0.0553	4
	E.Asia	RCOR	Flat	96	0.0032	0.0244	13
			Tilt	96	0.0044	0.0346	13
			OAT	96	0.0044	0.0335	13
	W.Siberia	ENE	Flat	55	0.0005	0.0076	7
			Tilt	55	0.0013	0.0158	8
			OAT	55	0.0010	0.0129	8
		ROAD	Flat	39	0.0005	0.0076	7
			Tilt	39	0.0012	0.0158	8

Table S4 Continued.

Period	Region	Sector	Panel setting	Area fractions (%)	Cleaning benefits (unitless)	PM soiling impacts (unitless)	Cleaning benefits (%)
			OAT	39	0.0010	0.0129	7
	E.C.Asia	ROAD	Flat	64	0.0011	0.0475	2
			Tilt	69	0.0024	0.0832	3
			OAT	67	0.0020	0.0743	3
		ENE	Flat	26	0.0007	0.0475	1
			Tilt	20	0.0014	0.0832	2
			OAT	22	0.0012	0.0743	2
MAM	S.Asia	RCOR	Flat	93	0.0076	0.0608	13
			Tilt	93	0.0075	0.0587	13
			OAT	93	0.0096	0.0738	13
	Tibetan-Plateau	RCOR	Flat	94	0.0055	0.0374	15
			Tilt	94	0.0056	0.0371	15
			OAT	94	0.0058	0.0342	17
	Arabian-Peninsula	ROAD	Flat	80	0.0027	0.1266	2
			Tilt	80	0.0029	0.1211	2
			OAT	80	0.0046	0.1553	3
	W.C.Asia	ROAD	Flat	89	0.0025	0.0731	3
			Tilt	89	0.0027	0.0674	4
			OAT	89	0.0038	0.0920	4
	E.Asia	RCOR	Flat	93	0.0044	0.0353	12
			Tilt	94	0.0045	0.0351	13
			OAT	93	0.0060	0.0447	13
	W.Siberia	ENE	Flat	55	0.0029	0.0289	10
			Tilt	56	0.0032	0.0294	11
			OAT	55	0.0043	0.0383	11
		ROAD	Flat	36	0.0019	0.0289	7
			Tilt	36	0.0021	0.0294	7
			OAT	36	0.0027	0.0383	7
	E.C.Asia	ROAD	Flat	67	0.0026	0.1067	2
			Tilt	67	0.0030	0.1038	3
			OAT	69	0.0040	0.1360	3
		RCOR	Flat	20	0.0019	0.1067	2
			Tilt	20	0.0022	0.1038	2
			OAT	18	0.0029	0.1360	2
JJA	S.Asia	RCOR	Flat	93	0.0036	0.0348	10
			Tilt	93	0.0033	0.0300	11
			OAT	93	0.0047	0.0448	10
	Tibetan-Plateau	RCOR	Flat	91	0.0047	0.0323	15
			Tilt	91	0.0043	0.0284	15
			OAT	91	0.0056	0.0338	17
	Arabian-Peninsula	ROAD	Flat	81	0.0026	0.1328	2
			Tilt	80	0.0025	0.1174	2
			OAT	80	0.0041	0.1636	3
	W.C.Asia	ROAD	Flat	84	0.0029	0.0898	3
			Tilt	84	0.0027	0.0721	4
			OAT	83	0.0043	0.1086	4
	E.Asia	RCOR	Flat	65	0.0015	0.0147	10
			Tilt	65	0.0013	0.0121	10
			OAT	65	0.0021	0.0192	11
		IND	Flat	19	0.0008	0.0147	6
			Tilt	19	0.0007	0.0121	6
			OAT	19	0.0012	0.0192	6
	W.Siberia	ENE	Flat	51	0.0033	0.0322	10
			Tilt	51	0.0028	0.0262	11
			OAT	52	0.0047	0.0427	11
		ROAD	Flat	36	0.0020	0.0322	6
			Tilt	36	0.0018	0.0262	7
			OAT	35	0.0029	0.0427	7
	E.C.Asia	ROAD	Flat	69	0.0024	0.1118	2
			Tilt	69	0.0024	0.0916	3

Table S4 Continued.

Period	Region	Sector	Panel setting	Area fractions (%)	Cleaning benefits (unitless)	PM soiling impacts (unitless)	Cleaning benefits (%)
			OAT	69	0.0035	0.1404	3
		RCOR	Flat	16	0.0015	0.1118	1
			Tilt	16	0.0015	0.0916	2
			OAT	14	0.0023	0.1404	2
SON	S.Asia	RCOR	Flat	93	0.0022	0.0236	9
			Tilt	93	0.0026	0.0268	10
			OAT	93	0.0032	0.0293	11
	Tibetan-Plateau	RCOR	Flat	91	0.0032	0.0212	15
			Tilt	91	0.0042	0.0264	16
			OAT	91	0.0031	0.0189	17
	Arabian-Peninsula	ROAD	Flat	71	0.0020	0.1131	2
			Tilt	75	0.0027	0.1329	2
			OAT	71	0.0044	0.1506	3
		ENE	Flat	29	0.0015	0.1131	1
			Tilt	25	0.0019	0.1329	1
			OAT	29	0.0033	0.1506	2
	W.C.Asia	ROAD	Flat	80	0.0018	0.0594	3
			Tilt	81	0.0026	0.0713	4
			OAT	82	0.0030	0.0788	4
	E.Asia	RCOR	Flat	51	0.0009	0.0095	9
			Tilt	53	0.0011	0.0122	9
			OAT	53	0.0012	0.0122	10
		IND	Flat	36	0.0008	0.0095	8
			Tilt	35	0.0010	0.0122	8
			OAT	35	0.0011	0.0122	9
	W.Siberia	ENE	Flat	53	0.0011	0.0128	8
			Tilt	52	0.0017	0.0188	9
			OAT	53	0.0017	0.0190	9
		ROAD	Flat	34	0.0008	0.0128	6
			Tilt	35	0.0014	0.0188	7
			OAT	34	0.0013	0.0190	7
	E.C.Asia	ROAD	Flat	64	0.0013	0.0652	2
			Tilt	67	0.0022	0.0874	3
			OAT	71	0.0024	0.0934	3
		ENE	Flat	17	0.0007	0.0652	1
			Tilt	21	0.0012	0.0874	1
			OAT	17	0.0013	0.0934	1

Table S5. The proportion of occupied areas, and regional area-weighted mean total benefits and PM total impacts and their ratios of sectors from which halving emissions provides the largest decadal and corresponding seasonal mean total benefits, in various regions of interest. For brevity, we sort sectors in descending order by their mean proportions of occupied areas of the three panels, and only keep to maximum two sectors whose cumulative mean proportions $\geq$ 75%. Please refer to Fig. S6 for definitions of regions that are presented here. Full definitions for source sectors are noncombustion agriculture (AGR), energy generation (ENE), industrial processes (IND), nonroad (NRTR) and on-road (ROAD) transportation, separate residential (RCOR), commercial (RCOC), and other (RCOO) sectors, waste (WST), solvent use (SLV), international shipping (SHP), and agricultural waster burning (AWB).

Period	Region	Sector	Panel setting	Area fractions (%)	Total benefits (unitless)	PM total impacts (unitless)	Total benefits (%)
Annual	S.Asia	RCOR	Flat	93	0.0059	0.0506	12
			Tilt	93	0.0065	0.0535	12

Table S5 Continued.

Period	Region	Sector	Panel setting	Area fractions (%)	Total benefits (unitless)	PM total impacts (unitless)	Total benefits (%)
			OAT	93	0.0078	0.0636	12
	E.Asia	RCOR	Flat	76	0.0035	0.0335	10
			Tilt	84	0.0043	0.0396	11
			OAT	79	0.0050	0.0454	11
	Tibetan-Plateau	RCOR	Flat	91	0.0046	0.0317	15
			Tilt	94	0.0054	0.0351	15
			OAT	94	0.0051	0.0315	16
	Arabian-Peninsula	ROAD	Flat	70	0.0028	0.1253	2
			Tilt	70	0.0033	0.1341	2
			OAT	75	0.0048	0.1605	3
		ENE	Flat	30	0.0021	0.1253	2
			Tilt	30	0.0025	0.1341	2
			OAT	25	0.0036	0.1605	2
	W.C.Asia	ROAD	Flat	84	0.0025	0.0722	3
			Tilt	84	0.0031	0.0757	4
			OAT	84	0.0038	0.0920	4
	W.Siberia	ENE	Flat	60	0.0021	0.0235	9
			Tilt	60	0.0026	0.0277	9
			OAT	63	0.0033	0.0337	10
		ROAD	Flat	32	0.0014	0.0235	6
			Tilt	33	0.0018	0.0277	7
			OAT	31	0.0022	0.0337	6
	E.C.Asia	ROAD	Flat	74	0.0022	0.0894	2
			Tilt	74	0.0030	0.1004	3
			OAT	74	0.0034	0.1197	3
		ENE	Flat	17	0.0013	0.0894	1
			Tilt	17	0.0018	0.1004	2
			OAT	17	0.0021	0.1197	2
DJF	S.Asia	RCOR	Flat	93	0.0064	0.0480	13
			Tilt	93	0.0084	0.0617	14
			OAT	93	0.0093	0.0628	15
	E.Asia	RCOR	Flat	96	0.0049	0.0362	13
			Tilt	96	0.0075	0.0562	13
			OAT	96	0.0072	0.0520	14
	Tibetan-Plateau	RCOR	Flat	94	0.0036	0.0228	16
			Tilt	94	0.0057	0.0346	16
			OAT	94	0.0043	0.0251	17
	Arabian-Peninsula	ROAD	Flat	68	0.0022	0.1004	2
			Tilt	68	0.0034	0.1363	3
			OAT	70	0.0043	0.1428	3
		ENE	Flat	32	0.0017	0.1004	2
			Tilt	32	0.0025	0.1363	2
			OAT	30	0.0032	0.1428	2
	W.C.Asia	ROAD	Flat	85	0.0016	0.0436	4
			Tilt	85	0.0029	0.0669	4
			OAT	85	0.0026	0.0619	4
	W.Siberia	ENE	Flat	60	0.0006	0.0088	7
			Tilt	62	0.0016	0.0208	8
			OAT	60	0.0011	0.0158	7
		ROAD	Flat	38	0.0006	0.0088	7
			Tilt	37	0.0015	0.0208	7
			OAT	38	0.0011	0.0158	7
	E.C.Asia	ROAD	Flat	70	0.0013	0.0511	3
			Tilt	70	0.0030	0.0919	3
			OAT	70	0.0024	0.0805	3
		RCOR	Flat	21	0.0010	0.0511	2
			Tilt	21	0.0023	0.0919	3
			OAT	21	0.0019	0.0805	2
MAM	S.Asia	RCOR	Flat	93	0.0091	0.0755	12
			Tilt	93	0.0091	0.0734	12

Table S5 Continued.

Period	Region	Sector	Panel setting	Area fractions (%)	Total benefits (unitless)	PM total impacts (unitless)	Total benefits (%)
			OAT	93	0.0115	0.0916	13
	E.Asia	RCOR	Flat	76	0.0054	0.0522	10
			Tilt	78	0.0056	0.0529	11
			OAT	74	0.0075	0.0675	11
	Tibetan-Plateau	RCOR	Flat	94	0.0060	0.0425	14
			Tilt	94	0.0062	0.0425	15
			OAT	94	0.0063	0.0396	16
	Arabian-Peninsula	ROAD	Flat	80	0.0031	0.1351	2
			Tilt	80	0.0033	0.1297	3
			OAT	80	0.0050	0.1638	3
	W.C.Asia	ROAD	Flat	88	0.0030	0.0812	4
			Tilt	88	0.0032	0.0762	4
			OAT	88	0.0044	0.1015	4
	W.Siberia	ENE	Flat	57	0.0032	0.0335	9
			Tilt	59	0.0036	0.0359	10
			OAT	60	0.0048	0.0458	10
		ROAD	Flat	34	0.0021	0.0335	6
			Tilt	34	0.0024	0.0359	7
			OAT	32	0.0030	0.0458	7
	E.C.Asia	ROAD	Flat	69	0.0030	0.1160	3
			Tilt	74	0.0035	0.1145	3
			OAT	74	0.0045	0.1470	3
		RCOR	Flat	13	0.0021	0.1160	2
			Tilt	13	0.0025	0.1145	2
			OAT	13	0.0032	0.1470	2
JJA	S.Asia	RCOR	Flat	93	0.0044	0.0456	10
			Tilt	93	0.0040	0.0394	10
			OAT	93	0.0058	0.0591	10
	E.Asia	RCOR	Flat	45	0.0022	0.0261	8
			Tilt	45	0.0018	0.0217	8
			OAT	43	0.0031	0.0356	9
		IND	Flat	43	0.0020	0.0261	8
			Tilt	43	0.0017	0.0217	8
			OAT	46	0.0029	0.0356	8
	Tibetan-Plateau	RCOR	Flat	91	0.0052	0.0369	14
			Tilt	91	0.0047	0.0324	15
			OAT	91	0.0061	0.0393	16
	Arabian-Peninsula	ROAD	Flat	70	0.0032	0.1460	2
			Tilt	70	0.0031	0.1289	2
			OAT	71	0.0049	0.1785	3
		ENE	Flat	30	0.0024	0.1460	2
			Tilt	30	0.0023	0.1289	2
			OAT	29	0.0037	0.1785	2
	W.C.Asia	ROAD	Flat	83	0.0033	0.0992	3
			Tilt	83	0.0031	0.0800	4
			OAT	83	0.0048	0.1191	4
	W.Siberia	ENE	Flat	58	0.0036	0.0368	10
			Tilt	59	0.0031	0.0307	10
			OAT	62	0.0053	0.0505	10
		ROAD	Flat	31	0.0022	0.0368	6
			Tilt	31	0.0019	0.0307	6
			OAT	30	0.0031	0.0505	6
	E.C.Asia	ROAD	Flat	71	0.0028	0.1196	2
			Tilt	74	0.0027	0.0986	3
			OAT	72	0.0040	0.1505	3
		ENE	Flat	20	0.0017	0.1196	1
			Tilt	17	0.0016	0.0986	2
			OAT	19	0.0025	0.1505	2
SON	S.Asia	RCOR	Flat	93	0.0035	0.0335	11
			Tilt	93	0.0043	0.0394	11

Table S5 Continued.

Period	Region	Sector	Panel setting	Area fractions (%)	Total benefits (unitless)	PM total impacts (unitless)	Total benefits (%)
			OAT	93	0.0048	0.0409	12
	E.Asia	IND	Flat	44	0.0016	0.0198	8
			Tilt	39	0.0022	0.0275	8
			OAT	42	0.0023	0.0264	9
		RCOR	Flat	33	0.0016	0.0198	8
			Tilt	35	0.0022	0.0275	8
			OAT	32	0.0023	0.0264	9
	Tibetan-Plateau	RCOR	Flat	91	0.0036	0.0246	15
			Tilt	91	0.0048	0.0311	16
			OAT	91	0.0036	0.0221	16
	Arabian-Peninsula	ROAD	Flat	60	0.0026	0.1197	2
			Tilt	63	0.0034	0.1414	2
			OAT	65	0.0049	0.1571	3
		ENE	Flat	40	0.0022	0.1197	2
			Tilt	37	0.0029	0.1414	2
			OAT	35	0.0040	0.1571	3
	W.C.Asia	ROAD	Flat	77	0.0022	0.0648	3
			Tilt	78	0.0032	0.0797	4
			OAT	78	0.0035	0.0856	4
	W.Siberia	ENE	Flat	63	0.0012	0.0148	8
			Tilt	64	0.0021	0.0235	9
			OAT	64	0.0020	0.0226	9
		ROAD	Flat	31	0.0009	0.0148	6
			Tilt	30	0.0016	0.0235	7
			OAT	30	0.0015	0.0226	6
	E.C.Asia	ROAD	Flat	76	0.0016	0.0707	2
			Tilt	72	0.0028	0.0966	3
			OAT	74	0.0028	0.1007	3
		ENE	Flat	20	0.0010	0.0707	1
			Tilt	20	0.0016	0.0966	2
			OAT	20	0.0017	0.1007	2

References

(1) Driemel, A.; Augustine, J.; Behrens, K.; Colle, S.; Cox, C.; Cuevas-Agulló, E.; Denn, F. M.; Duprat, T.; Fukuda, M.; Grobe, H.; Haeffelin, M.; Hodges, G.; Hyett, N.; Ijima, O.; Kallis, A.; Knap, W.; Kustov, V.; Long, C. N.; Longenecker, D.; Lupi, A.; Maturilli, M.; Mimouni, M.; Ntsangwane, L.; Ogihara, H.; Olano, X.; Olefs, M.; Omori, M.; Passamani, L.; Pereira, E. B.; Schmithüsen, H.; Schumacher, S.; Sieger, R.; Tamlyn, J.; Vogt, R.; Vuilleumier, L.; Xia, X.; Ohmura, A.; König-Langlo, G. Baseline Surface Radiation Network (BSRN): structure and data description (1992–2017). *Earth System Science Data* **2018**, *10*, 1491–1501.

(2) Giles, D. M.; Sinyuk, A.; Sorokin, M. G.; Schafer, J. S.; Smirnov, A.; Slutsker, I.; Eck, T. F.; Holben, B. N.; Lewis, J. R.; Campbell, J. R.; Welton, E. J.; Korkin, S. V.; Lyapustin, A. I. Advancements in the Aerosol Robotic Network (AERONET) Version 3 database – automated near-real-time quality control algorithm with improved cloud screening for Sun photometer aerosol optical depth (AOD) measurements. *Atmospheric Measurement Techniques* **2019**, *12*, 169–209.

(3) Zhang, Q.; Zheng, Y.; Tong, D.; Shao, M.; Wang, S.; Zhang, Y.; Xu, X.; Wang, J.; He, H.; Liu, W.; Ding, Y.; Lei, Y.; Li, J.; Wang, Z.; Zhang, X.; Wang, Y.; Cheng, J.; Liu, Y.; Shi, Q.; Yan, L.; Geng, G.; Hong, C.; Li, M.; Liu, F.; Zheng, B.; Cao, J.; Ding, A.; Gao, J.; Fu, Q.; Huo, J.; Liu, B.; Liu, Z.; Yang, F.; He, K.; Hao, J. Drivers of improved $PM_{2.5}$ air quality in China from 2013 to 2017. *Proceedings of the National Academy of Sciences* **2019**, *116*, 24463–24469.

(4) Wavelength dependence of the optical depth of biomass burning, urban, and desert dust aerosols. *Journal of Geophysical Research: Atmospheres* **1999**, *104*, 31333–31349.

(5) Latimer, R. N. C.; Martin, R. V. Interpretation of measured aerosol mass scattering efficiency over North America using a chemical transport model. *Atmospheric Chemistry and Physics* **2019**, *19*, 2635–2653.

(6) Ridley, D. A.; Heald, C. L.; Ford, B. North African dust export and deposition: A satellite and model perspective. *Journal of Geophysical Research: Atmospheres* **2012**, *117*, D02202.

(7) Hammer, M. S.; van Donkelaar, A.; Li, C.; Lyapustin, A.; Sayer, A. M.; Hsu, N. C.; Levy, R. C.; Garay, M. J.; Kalashnikova, O. V.; Kahn, R. A.; Brauer, M.; Apte, J. S.; Henze, D. K.; Zhang, L.; Zhang, Q.; Ford, B.; Pierce, J. R.; Martin, R. V. Global Estimates and Long-Term Trends of Fine Particulate Matter Concentrations (1998–2018). *Environmental Science & Technology* **2020**, *54*, 7879–7890.

(8) Miao, R.; Chen, Q.; Zheng, Y.; Cheng, X.; Sun, Y.; Palmer, P. I.; Shrivastava, M.; Guo, J.; Zhang, Q.; Liu, Y.; Tan, Z.; Ma, X.; Chen, S.; Zeng, L.; Lu, K.; Zhang, Y. Model bias in simulating major chemical components of $PM_{2.5}$ in China. *Atmospheric Chemistry and Physics* **2020**, *20*, 12265–12284.

(9) National Energy Administration. Statistics of photovoltaic power generation in 2019. `http://www.nea.gov.cn/2020-02/28/c_138827923.htm` (accessed 2022-05-03).

(10) Energy and Resources Institute. State-wise installed capacity of Grid Interactive Solar Power as on 31.12.2019. `http://www.terienvis.nic.in/index3.aspx?sslid=9575&subsublinkid=2709&langid=1&mid=1` (accessed 2022-05-03).

(11) Sweerts, B.; Pfenninger, S.; Yang, S.; Folini, D.; van der Zwaan, B.; Wild, M. Estimation of losses in solar energy production from air pollution in China since 1960 using surface radiation data. *Nature Energy* **2019**, *4*, 657–663.

(12) GlobalPetrolPrices.com. Electricity prices for households, September 2020. `https://www.globalpetrolprices.com/electricity_prices/` (accessed 2021-10-10).

(13) Iturbide, M.; Gutiérrez, J. M.; Alves, L. M.; Bedia, J.; Cerezo-Mota, R.; Cimadevilla, E.; Cofiño, A. S.; Di Luca, A.; Faria, S. H.; Gorodetskaya, I. V.; Hauser, M.; Herrera, S.; Hennessy, K.; Hewitt, H. T.; Jones, R. G.; Krakovska, S.; Manzanas, R.; Martínez-Castro, D.; Narisma, G. T.; Nurhati, I. S.; Pinto, I.; Seneviratne, S. I.; van den Hurk, B.; Vera, C. S. An update of IPCC climate reference regions for subcontinental analysis of climate model data: definition and aggregated datasets. *Earth System Science Data* **2020**, *12*, 2959–2970.

(14) Bergin, M. H.; Ghoroi, C.; Dixit, D.; Schauer, J. J.; Shindell, D. T. Large Reductions in Solar Energy Production Due to Dust and Particulate Air Pollution. *Environmental Science & Technology Letters* **2017**, *4*, 339–344.

(15) Li, X.; Mauzerall, D. L.; Bergin, M. H. Global reduction of solar power generation efficiency due to aerosols and panel soiling. *Nature Sustainability* **2020**, *3*, 720–727.
